# A Simplified Crossing Fiber Model in Diffusion Weighted Imaging

**DOI:** 10.3389/fnins.2019.00492

**Published:** 2019-05-22

**Authors:** Sheng Yang, Kaushik Ghosh, Ken Sakaie, Satya S. Sahoo, Sarah J. Ann Carr, Curtis Tatsuoka

**Affiliations:** ^1^Department of Population and Quantitative Health Sciences, Case Western Reserve University, Cleveland, OH, United States; ^2^Department of Mathematical Sciences, University of Nevada, Las Vegas, NV, United States; ^3^Department of Diagnostic Radiology, Cleveland Clinic Foundation, Cleveland, OH, United States; ^4^Department of Neuroimaging, King's College London, London, United Kingdom; ^5^Department of Neurology, Case Western Reserve University, Cleveland, OH, United States

**Keywords:** Ball-and-Stick model, Bayesian, dMRI, crossing fibers, simplified model

## Abstract

Diffusion MRI (dMRI) is a vital source of imaging data for identifying anatomical connections in the living human brain that form the substrate for information transfer between brain regions. dMRI can thus play a central role toward our understanding of brain function. The quantitative modeling and analysis of dMRI data deduces the features of neural fibers at the voxel level, such as direction and density. The modeling methods that have been developed range from deterministic to probabilistic approaches. Currently, the Ball-and-Stick model serves as a widely implemented probabilistic approach in the tractography toolbox of the popular FSL software package and FreeSurfer/TRACULA software package. However, estimation of the features of neural fibers is complex under the scenario of two crossing neural fibers, which occurs in a sizeable proportion of voxels within the brain. A Bayesian non-linear regression is adopted, comprised of a mixture of multiple non-linear components. Such models can pose a difficult statistical estimation problem computationally. To make the approach of Ball-and-Stick model more feasible and accurate, we propose a simplified version of Ball-and-Stick model that reduces parameter space dimensionality. This simplified model is vastly more efficient in the terms of computation time required in estimating parameters pertaining to two crossing neural fibers through Bayesian simulation approaches. Moreover, the performance of this new model is comparable or better in terms of bias and estimation variance as compared to existing models.

## Introduction

Recent advances in neuroscience technologies to study brain connectivity are providing new techniques to better understand the role of both structural and functional networks in complex neurological disorders (Bargmann and Newsome, 2014; Baliyan et al., 2016). A fundamental goal of modern neuroscience is to understand how different cortical regions interact with one another to produce observed behavior (Behrens et al., 2003; Yang et al., 2016). Diffusion MRI (dMRI) allows for identifying anatomical connections in the living human brain. These connections form the substrate for information transfer between remote brain regions and are therefore central to our understanding of brain function.

Fiber tractrography, inferred from dMRI, offers the only way to study brain structural connectivity non-invasively *in vivo* to reconstruct nerve fiber tracts. It has become an important tool in the study of a wide range of diseases affecting the brain, as it allows us to probe the shape and integrity of the white-matter pathways that connect the functionally-related cortical and subcortical regions (Yendiki et al., 2011, 2016). A lot of research has focused on discovering an approach using parametrized models for extracting tissue structural information from dMRI data.

An early, widely accepted model is the diffusion tensor model (DTI), which derives the orientation of a fiber within a voxel, the smallest analytic unit (Basser et al., 1994; Mori and van Zijl, 2002). Even though it is a robust model when there is only a single major diffusion direction in a given voxel, it has the inherent limitation when voxels contain more than one fiber pathways with distinct orientations. Voxels with crossing fibers account for more than 60% of the total voxels across the brain (Jeurissen et al., 2013). In order to handle the more complex situation of multiple crossing fibers within a voxel, a number of alternative models have been suggested, such as multiple tensor fitting (Tuch et al., 2002), Ball-and-Stick model (Behrens et al., 2003, 2007; Hosey et al., 2005; Jbabdi et al., 2007), spherical deconvolution (Tournier et al., 2004; Anderson, 2005) and Q-ball imaging (Hess et al., 2006). The methods each have their limitations. Spherical deconvolution attempts to estimate the fiber orientation directly and assumes a fiber response function. The Ball-and-Stick method models axons as impermeable cylinders of a single radius and assumes isotropic diffusion outside the axon in the extracellular compartments. Although the spatial profiles from both the Ball-and-Stick method and spherical deconvolution appear similar, conceptually there is quite a difference. Geometric features of the signal itself are used to constrain the Ball-and-Stick model. Extensions to improve the Ball-and-Stick model have been proposed, such as the ball and racket method described by Sotiropoulos et al. (2012). This incorporates a Bingham distribution to model fanning of the tracts, common in the corona radiatus. An alternative to the Ball-and-Stick method is the composite hindered and restricted model of diffusion (CHAMRED) developed by Assaf et al. (2004). It is a hybrid model that uses diffusion tensors to model the extracellular diffusion as a hindered space and the intracellular diffusion is modeled as a restricted cylinder. This method has also been extended by Zhang et al. (2011) who apply a Watson distribution (a spherical analog of a Gaussian distribution) to the CHAMRED methodology to estimate the diameters of axons. The technique is limited by the assumption that axons are orientated in a single dominant direction excluding the possibility of detecting crossing or bending fibers. Here, we study and refine the Bayesian inference framework for the Ball-and-Stick model, as it is widely implemented in the tractography toolbox of the popular FSL software package (University of Oxford) and FreeSurfer/TRACULA software (Massachusetts General Hospital and Harvard Medical School).

In the situation of two crossing fibers, we discover from simulation analyses that the Ball-and-Stick model faces significant challenges in estimation of the fiber-related parameters, when we apply the Bayesian framework proposed by Behrens (Behrens et al., 2007). For example, convergence of the sampling scheme of Markov Chain Monte Carlo (MCMC) procedure can be slow in the Ball-and-Stick model, because the sampling and simulation is conducted in a high-dimensional parameter space, and within a computationally challenging non-linear regression modeling context. This can lead to long sampling chains without convergence. The study of a simplified version of a Ball-and-Stick model is motivated by the need for reducing complexity of the non-linear regression model, more robust estimation of the parameters related to neural fibers, and improved efficiency in MCMC simulations.

Focus on the two-fiber model has practical justification. It is difficult for 3 or more fiber directions to be well separated, except under limited scenarios, and even then, it can be difficult to distinguish 3 fiber models from isotropy. Hence, accurately estimating 3-fiber models is not often feasible, especially with a limited number of gradients.

We propose a simplified version of Ball-and-Stick through a two-stage approach. First we develop a set of simultaneous equations for estimating “nuisance” parameters (i.e., those that are not directly related to the features of neural fibers). Further, we simplify this non-linear regression model through rotation by exploiting geometric properties of the surface of the intensity model shape.

This simplified model is validated by comparing fiber-related parameter estimation with the full Ball-and-Stick model in an efficient, adaptive MCMC estimation framework. Simulation analysis and an *in-vivo* human data example demonstrate the improved computational efficiency and estimation accuracy of the proposed simplified model.

We also explore how to dramatically improve computational efficiency by applying voxel-specific dynamic stopping rules for MCMC sampling based on convergence monitoring statistics. Finally, methods for determining between one- vs. two-fiber model fit and switching models within a single MCMC run are studied. Together, our approaches hold the promise for vast improvements in computational times for generating probabilistic fiber tracking, which currently is extremely slow.

## Materials and Methods

### Introduction of Ball-and-Stick Model

A local partial volume model for diffusion signal attenuation (e.g., restriction of water diffusion), the Ball-and-Stick model, was proposed by Behrens et al. (2003). The model relates the local fiber structure to the diffusion signal by assuming different components within each voxel. The diffusion-weighted MR signal (S_i_) is split into multiple components for each fiber orientation, and a single isotropic component.

The Ball-and-Stick model is used to retrieve component-specific information (free and restricted diffusion) from the signal decay in diffusion MRI experiments. The fully parameterized model can be fitted to dMRI data (similar sampling scheme as for DTI). This model is notably implemented in BedPostX and available in FSL (http://fsl.fmrib.ox.ac.uk/fsl/fslwiki/FDT).

### Formula and Notation of Ball-and-Stick Model

The predicted signal for each diffusion-weighted measurement at each voxel is:

(1) $$\begin{array}{l} S_{i}=S_{0}[\left(1-\overset{L}{\underset{k=1}{\sum }}f_{k}\right)\exp\left(-b_{i}d\right)\\ \;+\overset{L}{\underset{k=1}{\sum }}f_{k}\exp\left(-b_{i}dr_{i}^{T}R_{k}AR_{k}^{T}r_{i}\right)] \end{array}$$

where *S*_*i*_ is the observed signal intensity along the *i-th* diffusion-weighting gradient*; S*_0_ is the signal intensity without diffusion gradients; *b*_*i*_ is the diffusion weighting factor for the *i-th* gradient; *f*_*k*_ is the volume fraction of the *k-*th fiber; (1-$\overset{L}{\underset{k=1}{\sum }}{\;f}_{k}$) is the volume fraction for isotropic tissue; *d* is a diffusivity constant; r_i_ is the directional unit vector of the *i-th* diffusion-weighting gradient*; R*_*k*_is a rotation matrix depending on *(*θ_*k*_, ϕ_*k*_*)*: the spherical coordinates for elevation angle and azimuth angle, respectively.

$$\begin{array}{lll} R_{k} & = & \left[\begin{array}{c} \begin{array}{ccc} \cos\theta_{k}\cos\varphi_{k} & \;-\sin\theta_{k} & \;\sin\theta_{k}\cos\varphi_{k} \end{array}\\ \begin{array}{ccc} \cos\theta_{k}\sin\varphi_{k} & \;\cos\varphi_{k} & \;\sin\theta_{k}\sin\varphi_{k} \end{array}\\ \begin{array}{ccc} \sin\theta_{k} & \;0 & \;-\cos\theta_{k} \end{array} \end{array}\right]\\ A & = & \left[\begin{array}{c} \begin{array}{c} \begin{array}{ccc} 1 & 0 & 0 \end{array}\\ \begin{array}{ccc} 0 & 0 & 0 \end{array} \end{array}\\ \begin{array}{ccc} 0 & 0 & 0 \end{array} \end{array}\right] \end{array}$$

In the model, the noise is modeled separately for each voxel as independent identically distributed Gaussians with a mean of zero and standard deviation of σ across acquisitions (Behrens et al., 2007). Note that the commonly-used noise distribution for MRI signal will converge to a Gaussian distribution asymptotically (Gudbjartsson and Patz, 1995) in the context of high signal to noise ratio.

In Equation 1, the formula consists of two major components. The first component in the bracket, the “Ball” component,${\;S}_{0}\left(1-\overset{L}{\underset{k=1}{\sum }}{\;f}_{k}\right)\exp\left({-b}_{i}d\right),\;$represents the part of the *i-th* diffusion signal intensity attributable to the isotropy (e.g., free water). The second component, the “Stick” component, $S_{0}\overset{L}{\underset{k=1}{\sum }}{\;f}_{k}\exp\left(-b_{i}dr_{i}^{T}R_{k}AR_{k}^{T}r_{i}^{T}\right)$, denotes the contribution attributable to fiber tracts.

Instead of estimating all the parameters at once with non-linear regression under an MCMC framework, a proposed simplified model will reduce the number of parameters estimated through MCMC and non-linear regression. Estimation approaches will be derived for *d* and fiber-specific parameters (e.g., $\sum f_{k}$, θ). As is commonly applied, note that a good estimator for *S*_0_ is the average of the diffusion signal values when *b*_*i*_ is equal to 0.

### Reformulation of the Ball-and-Stick Model

The reformulation of the Ball-and-Stick model concentrates on the “Stick” component of diffusion attenuation, $S_{0}\overset{L}{\underset{k=1}{\sum }}{\;f}_{k}\exp\left(-b_{i}dr_{i}^{T}R_{k}AR_{k}^{T}r_{i}^{T}\right)\;$(Behrens et al., 2003; Dell'Acqua et al., 2007). Recall that the parameters that reflect the k-th neural fiber orientation is (θ_k_, φ_k_) in spherical coordinate system, where θ refers to the elevation angle, which measures the angular separation between the corresponding directional vector and XY-plane; φ refers to the azimuthal angle, which measures the angular separation between directional vector and XZ-plane. It can also be denoted in the Cartesian coordinate system, so that the notation for the corresponding unit vector direction is t_k_ = [cosθkcosφk cosθksinφk sinθk]Tor [ti,x ti,y ti,z]T. Also, let *r*_*i*_ be the unit directional vector of the *i-th* gradient, ${\left[\begin{array}{c} r_{i,x}r_{i,y}r_{i,z} \end{array}\right]}^{\text{T}}$ = ${\left[\begin{array}{c} cos\theta_{i}cos\varphi_{i}cos\theta_{i}sin\varphi_{i}sin\theta_{i} \end{array}\right]}^{\text{T}}$.

The dot product *r*_i_ · *t*_*k*_represents the projection of *i-th* gradient onto the direction of the *k-th* neural fiber. Denote Δ_ik_ as the angular separation of these two directions, then *r*_i_ · *t*_*k*_ = cos(Δ_ik_) | *r*_i_ | | *t*_*k*_ | = cos(Δ_ik_). Generally, the expression of $\exp\left(-b_{i}dr_{i}^{T}R_{k}AR_{k}^{T}r_{i}\right)$ in Equation (1) can be reformulated as $\exp\left(-b_{i}dcos^{2}\Delta_{ik}\right)$, so that

(2) $$\begin{array}{r} r_{i}^{T}R_{k}AR_{k}^{T}r_{i}^{T}={\left(r_{\text{i}}•t_{k}\right)}^{2}={\text{cos}}^{2}\left(\Delta_{\text{ik}}\right) \end{array}$$

For example, when the *i-th* gradient is perpendicular to the *k-th* fiber, Δ_ik_ is equal to 90°. This leads to Equation (2) being equal to 0, and the diffusion signal reaches a maximum with respect to the fiber.

### Simplification of the Two-fiber Ball-and-Stick Model

In our discussions to follow, we focus on two- and one-fiber models, and assume that the b-value is constant across directions. Simplification is in relation to two fiber models.

#### Derivation of Estimators for Voxel-Specific Diffusivity and Sum of Volume Fractions

Spherical mean techniques enable us to generate an equation based on observed diffusion data, that also involves diffusivity coefficient *d* and the value of the sum of fiber volume fractions, ∑***f***_***k***_. Importantly, this is achieved while averaging out the effect due to fiber dispersion (Kaden et al., 2016), so that the equation should be fairly stable and minimally affected by noise. It is based on the insight that for a fixed *b*-value, the spherical mean of the diffusion signal over the gradient directions does not depend on the fiber orientation distribution. Specifically, the mean signal is invariant with respect to the specific fiber orientations within a voxel, when all other sequence parameters S_0_, *d, f*, and the diffusion weighting factor *b* ≥ 0, are fixed.

Suppose we have a fixed set of L fibers, and gradient directions are uniformly spread across the sphere. We then derive the expected intensity value in this setting, which depends on *d* and ∑***f***_***k***_, but not the fiber orientations.

Let Δ represent the angular separation value between a gradient and fiber orientation. In the uniform gradient direction setting, the distribution function of values across the sphere is of the form f(Δ)$=\frac{1}{2}$sin Δ, across the range of values in (0, π). Note $\exp\left(-bdr_{i}^{T}R_{k}AR_{k}^{T}r_{i}\right)$ can be reformulated as $\exp\left(-bdcos^{2}\Delta_{ik}\right)$, as in Baliyan et al. (2016). By the integration across all possible gradient directions (i.e., Δ values) for each fiber orientation, we can derive Equation (3) in the setting of the Ball-and-Stick model Equation (1) to obtain an average theoretical mean intensity value that can be approximately equated with the empirical mean:

(3) $$\begin{array}{r} {\bar{S}}_{i}\;≅\;S_{0}\left[\left(1-\overset{L}{\underset{k=1}{\sum }}f_{k}\right)\exp\left(-b_{i}d\right)+\overset{L}{\underset{k=1}{\sum }}f_{k}\frac{\sqrt{\pi }Erf\left(\sqrt{b_{i}d}\right)}{2\sqrt{b_{i}d}}\right] \end{array}$$

given that

$$\begin{array}{l} E\left[\exp\left(-b_{i}dcos^{2}\Delta \right)\right]=\overset{\pi }{\underset{\Delta =0}{\int }}\exp\left(-b_{i}dcos^{2}\Delta \right)f\left(\Delta \right)\text{d}\Delta \\ \;=\frac{\sqrt{\pi }Erf\left(\sqrt{b_{i}d}\right)}{2\sqrt{b_{i}d}} \end{array}$$

and

$$\begin{array}{l} Erf\left(x\right)=\frac{1}{\sqrt{\pi }}\overset{x}{\underset{-x}{\int }}e^{{-t}^{2}}dt \end{array}$$

Note that the spherical mean of diffusion intensity derived in Equation (3) does not depend on the fiber orientations. We will thus use Equation (3) as a basis for estimating parameters, ∑***f***_***k***_ and *d*, in the Ball-Stick model outside of the non-linear regression framework. For that, we still need a second equation for estimation.

#### Estimating the Directionality of the Longitudinal Axis

**Figure 1:**
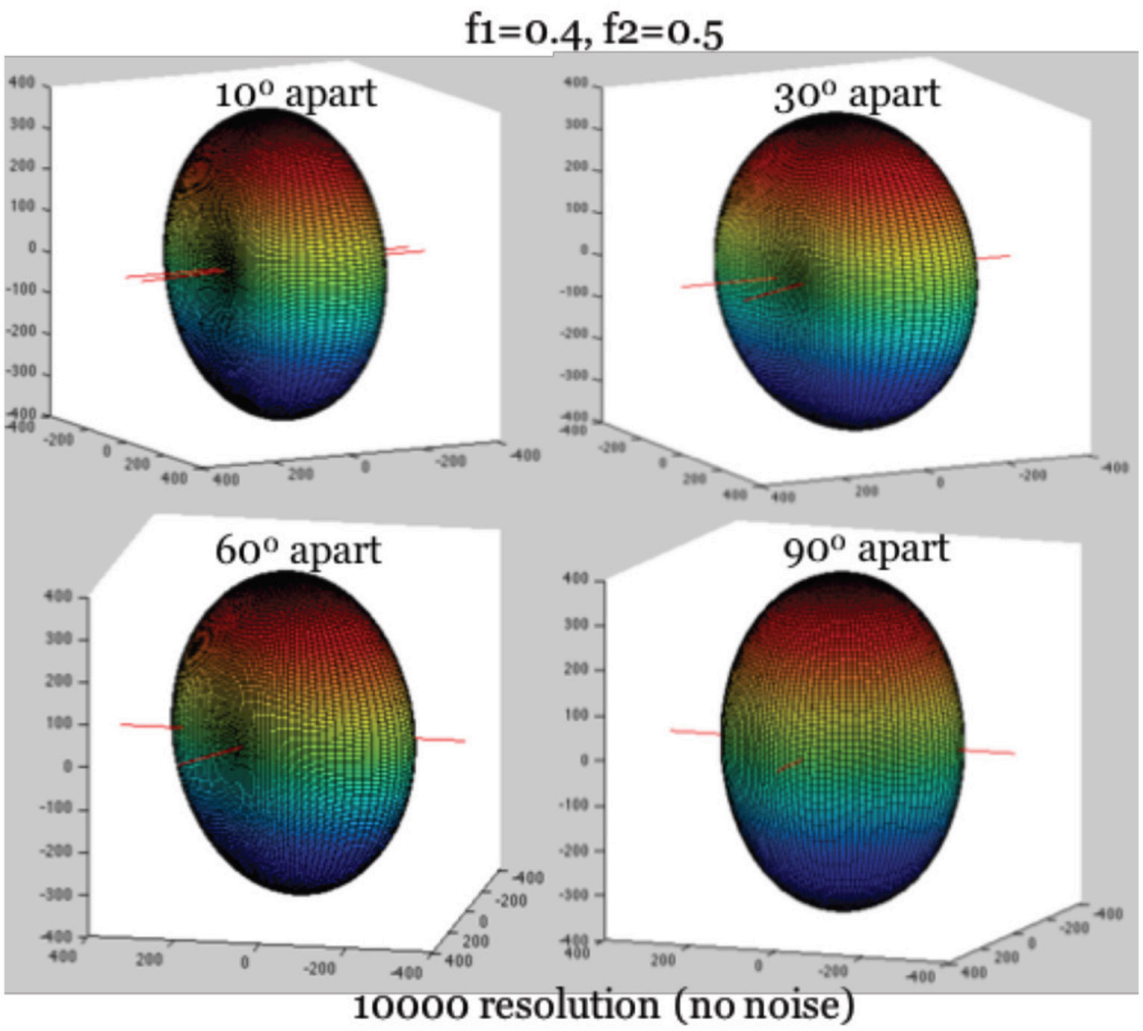
True diffusion signal shape with the longitudinal axis aligning with Z-axis. Note: The hyperplane containing two fibers are on the X-Y plane. The largest diffusion signal is always in the direction of longitudinal axis.

To simplify the estimation of the Ball-and-Stick model (Equation 1), we rely on a feature of the diffusion intensities that becomes clear as one visualizes their corresponding surface of intensities, as in Figure 1. Given a voxel containing two fibers, we define the *longitudinal axis* to be the axis perpendicular to the two fibers, and denote it by r_*i*_. The identification of r_*i*_ is critical for deriving a second simultaneous equation for estimation. It captures two major geometric features of the 3D surface of diffusion intensity values (see Figure 1): (1) The maximum of diffusion-weighted signal lies in the direction of r_*i*_. (2) Knowing its direction can be used to align the hyperplane of two neural fibers onto the X-Y plane through rotation, which can further reduce the complexity of angular parameterization of the orientations of the two fibers. Use of the first feature allows us to derive a needed second simultaneous equation for ∑***f***_***k***_ and *d*.

First, we identify the direction associated with the maximum of the diffusion-weighted signal, the orientation of r_*i*_. Since r_*i*_ is perpendicular to both fibers simultaneously, the angular separation between r_*i*_ and the two fibers, denoted as Δ_*i*1_ and Δ_*i*2_, are 90°, so that cos(Δ_*i*1_) = cos(Δ_*i*2_) = 0. Recall in Equation 1 that R_1_ is the rotation matrix of the orientation of the first neural fiber, and R_2_ is that of the second fiber. Then we have $r_{l}^{T}R_{1}AR_{1}^{T}r_{l}^{T}=\;$cos^2^(Δ_*i*1_) = 0, $r_{l}^{T}R_{2}AR_{2}^{T}r_{l}^{T}=$ cos^2^(Δ_*i*2_) = 0 based on Equation (2), where $r_{l}^{T}R_{1}AR_{1}^{T}r_{l}^{T}$ and $r_{l}^{T}R_{2}AR_{2}^{T}r_{l}^{T}$ reach their minimum simultaneously. The minimum of this expression further leads to the maximum possible intensity value on the surface, when it is substituted into Equation (1). Given *b* ≥ 0, we thus have:

(4) $$\begin{array}{r} \max\left(S\right)=S_{0}\left[\left(1-\overset{2}{\underset{k=1}{\sum }}f_{k}\right)\exp\left(-b_{i}d\right)+\overset{2}{\underset{k=1}{\sum }}f_{k}\right] \end{array}$$

By solving the set of Equations (3–4), we can obtain estimators for *d* and Σ* f*_*k*_ outside of the non-linear regression framework.

Secondly, the plane of two neural fibers can be mapped onto the X-Y plane, by rotating r_*i*_ onto the Z-axis. The rotation of r_*i*_ onto the Z-axis maps the gradient directions onto a new coordinate system. Given a known direction of r_*i*_, the alignment of r_*i*_ onto the Z-axis can be achieved by two steps: (1) Clockwise rotate Z-axis with angle φ_*i*_, such that the rotated longitudinal axis is on the X-Z plane, with an angular separation of θ_*i*_ relative to the X-Y plane; (2) Clockwise rotate Y-axis by (π/2- θ_*i*_), such that the previously rotated longitudinal axis will be mapped to the Z-axis. This procedure, a two-step matrix multiplication, results in a rotation matrix R_rot_. Note that we use the prime sign (′) to denote the mapping objects in the new coordinate system. For example, the directional vector of i-th gradient in the new coordinate system is denoted as ${{\text{r}}^{\prime }}_{\text{i}}$ = ${\left[\begin{array}{c} {cos\theta^{\prime }}_{i}{cos\varphi^{\prime }}_{i}{cos\theta^{\prime }}_{i}{sin\varphi^{\prime }}_{i}{sin\theta^{\prime }}_{i} \end{array}\right]}^{\text{T}}$or ${\left[\begin{array}{c} {r^{\prime }}_{i,x}\;{r^{\prime }}_{i,y}\;{r^{\prime }}_{i,z} \end{array}\right]}^{\text{T}}$. Similarly, the directional vector of the k-th neural fiber in the original coordinate system, t_k_ is mapped to the vector ${t^{\prime }}_{k}$ = ${\left[\begin{array}{c} {cos\theta^{\prime }}_{k}{cos\varphi^{\prime }}_{k}{cos\theta^{\prime }}_{k}{sin\varphi^{\prime }}_{k}{sin\theta^{\prime }}_{k} \end{array}\right]}^{\text{T}}$or ${\left[\begin{array}{c} {r^{\prime }}_{k,x}\;{r^{\prime }}_{k,y}\;{r^{\prime }}_{k,z} \end{array}\right]}^{\text{T}}$. The mapping into the new coordinate system is through the rotation matrix R_rot_:

$$\begin{array}{l} R_{\text{rot}}=\left[\begin{array}{ccc} {\cos\left(\frac{\pi }{2}-\theta_{l}\right)}_{\;} & 0 & -\sin\left(\frac{\pi }{2}-\theta_{l}\right)\\ 0 & 1 & 0\\ \sin\left(\frac{\pi }{2}-\theta_{l}\right) & 0 & \cos\left(\frac{\pi }{2}-\theta_{l}\right) \end{array}\right]\;\left[\begin{array}{ccc} cos\varphi_{l} & sin\varphi_{l} & 0\\ {-sin\varphi }_{l} & cos\varphi_{l} & 0\\ 0 & 0 & 1\\ \; \end{array}\right]\\ \;=\left[\begin{array}{ccc} sin\theta_{l}cos\varphi_{l} & sin\theta_{l}sin\varphi_{l} & {-cos\theta }_{l}\\ {-sin\phi }_{l} & cos\varphi_{l} & 0\\ cos\theta_{l}cos\varphi_{l} & cos\theta_{l}sin\varphi_{l} & sin\theta_{l} \end{array}\right]\; \end{array}$$

where r_*i*_ = [*cos*θ_*l*_*cos*φ_*l*_ *cos*θ_*l*_*sin*φ_*l*_ *sin*θ_*l*_]. Also note that we can map the vector in new coordinate system back to the original coordinate system:

$$\begin{array}{l} r_{i}\;=R_{rot\;}^{-1}{r^{\prime }}_{i}\;,\;t_{i}\;=R_{rot\;}^{-\;1}{t^{\prime }}_{i} \end{array}$$

With the hyperplane of two neural fibers being rotated onto the X-Y plane, the elevation angle ${\theta^{\prime }}_{\text{k}}$ relative to the X-Y plane is equal to 0. In this case, ${t^{\prime }}_{k}$ = ${\left[\begin{array}{c} {cos\varphi^{\prime }}_{k}{sin\varphi^{\prime }}_{k}\;0 \end{array}\right]}^{\text{T}}$, so that the orientation of the k-th fiber can be denoted by one parameter, φ′_*k*_. Thus, the complexity of denoting the orientation of two fibers can be reduced from four parameters (θ_1_, φ_1_, θ_2_, φ_2_) to two parameters (${\varphi^{\prime }}_{1}$, ${\varphi^{\prime }}_{2}$). After the acquisition of estimated values of (${\varphi^{\prime }}_{1}$, ${\varphi^{\prime }}_{2}$) in Equation 5, we can convert angles back into the original coordinate space.

#### Formula of Simplified Model

In the new coordinate system, the relative angular separation between two fibers will remain the same as that in the original coordinate system. For example, the angular separation between the i-th gradient and the k-th neural fiber in the original coordinate system, Δ_ik_, will be the same as $\Delta \prime {\text{i}}_{\text{k}}$ in the new system, such that cos^2^(Δ_ik_) = cos^2^(${\Delta^{\prime }}_{\text{ik}}$), which derives (*r*_i_ ·*t*_*k*_)^2^ = (*r*′ · *t*′_*k*_)^2^ based on Equation (2). Furthermore, we can reformulate the “Stick” component of diffusion intensity attributable to the fiber tracts, $\overset{2}{\underset{k=1}{\sum }}{\;f}_{k}\exp\left(-bdr_{i}^{T}R_{k}AR_{k}^{T}r_{i}^{T}\right)$, proportional to the baseline intensity S_0_, as

$$\begin{array}{l} \sum_{k=1}^{2}f_{k}\exp(-bdr_{i}^{T}R_{k}AR_{k}^{T}r_{i})\\ =f_{1}\exp(-bd{\left(r_{i}\cdot \;t_{1}\right)}^{2})+f_{2}\exp(-bd{\left(r_{i}\;\cdot \;t_{2}\right)}^{2})\\ =f_{1}\exp(-bd{\left(r_{i}^{\prime }\cdot \;t_{1}^{\prime }\right)}^{2})+f_{2}\exp(-bd{\left(r_{i}^{\prime }\cdot t_{2}^{\prime }\right)}^{2})\\ =f_{1}\exp(-bd{\left(r^{\prime }{\;}_{i,x}\cos\varphi_{1}{}^{\prime }+r^{\prime }{\;}_{i,y}\sin\varphi_{1}{}^{\prime }\right)}^{2})\\ +f_{2}\exp(-bd{\left(r^{\prime }{\;}_{i,x}\cos\varphi_{2}{}^{\prime }+r^{\prime }{\;}_{i,y}\sin\varphi_{2}{}^{\prime }\right)}^{2})\; \end{array}$$

Thus, in the new coordinate system, we obtain the simplified model by reformulating Equation 1 as

(5) $$\begin{array}{l} S_{i}={\hat{S}}_{0}[\left(1-\hat{\sum_{k=1}^{2}f_{k}}\right)\exp\left(-b_{i}\hat{d}\right)\\ \;+\;f_{1}\exp\;\left(-b\hat{d}{\left(r_{i,x}^{\prime }\cos\varphi_{1}^{\prime }+r_{i,y}^{\prime }-\sin\varphi_{1}^{\prime }\right)}^{2}\right)\\ \;+\left(\hat{\sum_{k=1}^{2}f_{k}}-f_{1}\right)\exp\;\left(-b_{i}\hat{d}\;{\left(r_{i,x}^{\prime }\cos\varphi_{2}^{\prime }-r_{i,y}^{\prime }\sin\varphi_{2}^{\prime }\right)}^{2}\right)] \end{array}$$

In the simplified model (Equation 5), we have demonstrated below that the nuisance parameters that are not directly related to the features of neural fibers, *S*_0_*, d*, Σ* f*_*k*_, *r*_*l*_, can be estimated outside the framework of MCMC estimation. We apply the “hat” sign to denote the estimators of parameters (e.g., $\hat{S_{0}}$, $\hat{d}$, $\hat{\Sigma {\;f}_{k}}$, $\hat{r_{l}}$). Thus, the number of parameters can be reduced from 9 in the full Ball-and-Stick model (Equation 1) to 4 in the simplified model (Equation 5). As we will establish, the simplification of the non-linear regression model has important ramifications for computational efficiency and can also lead to improved statistical accuracy in fiber-related parameter estimation.

### Estimation of the Simplified Model

#### Use of Spatially Smoothed Data

The purpose of spatial smoothing is to eliminate the effect of noise in the intensity values through spatially weighted averaging. Similar to the notation in the Ball-and-Stick model (Equation 1), *S*_*i*_ denotes the signal intensity along the i-th gradient direction in Equation (6). For illustration, we assume 64 observed gradient directions. Note that the estimation of nuisance parameters is prone to the effect of noise from the diffusion intensity measurement. To have a robust estimator of nuisance parameters, we propose smoothing each data point through a von-Mises smoothing kernel, centered on the respective gradient direction. von-Mises distributions are used in directional data applications (see Equation 6) and are analogous to wrapped normal distributions for angular data. Let x_ij_ denote the angular distance between directions i and j. *S*_*j, smooth*_ is the smoothed signal intensity for the gradient direction j. The denominator is to assure that the weights applied to the *S*_*i*_ sum to 1.

(6) $$\begin{array}{r} S_{j,smooth}=\frac{\sum_{i=1}^{64}f_{vonMises}\left(x_{ij},\kappa \right)S_{i}}{\sum_{i=1}^{64}f_{vonMises}\left(x_{ij},\kappa \right)} \end{array}$$

where

$$\begin{array}{l} f_{vonMises}\left(x_{ij},\kappa \right)=\frac{e^{\kappa cos\;x_{ij}}}{2\pi I_{0}\left(\kappa \right)}, \end{array}$$

and I_0_(κ) is the modified Bessel function of order 0.

In our simulation analysis, the gradient directions are 64 evenly distributed directions, as in Jones et al. (2002). We will demonstrate that spatially smoothing the diffusion data derived from noisy observed data can improve estimation of the nuisance parameters, if a proper smoothing kernel is applied. The concentration or “spread” of a von-Mises distribution is determined by a kappa (κ) parameter. It is thus of interest to identify appropriate κ values, which control the extent of smoothing, that ultimately improve estimation.

**Figure 2:**
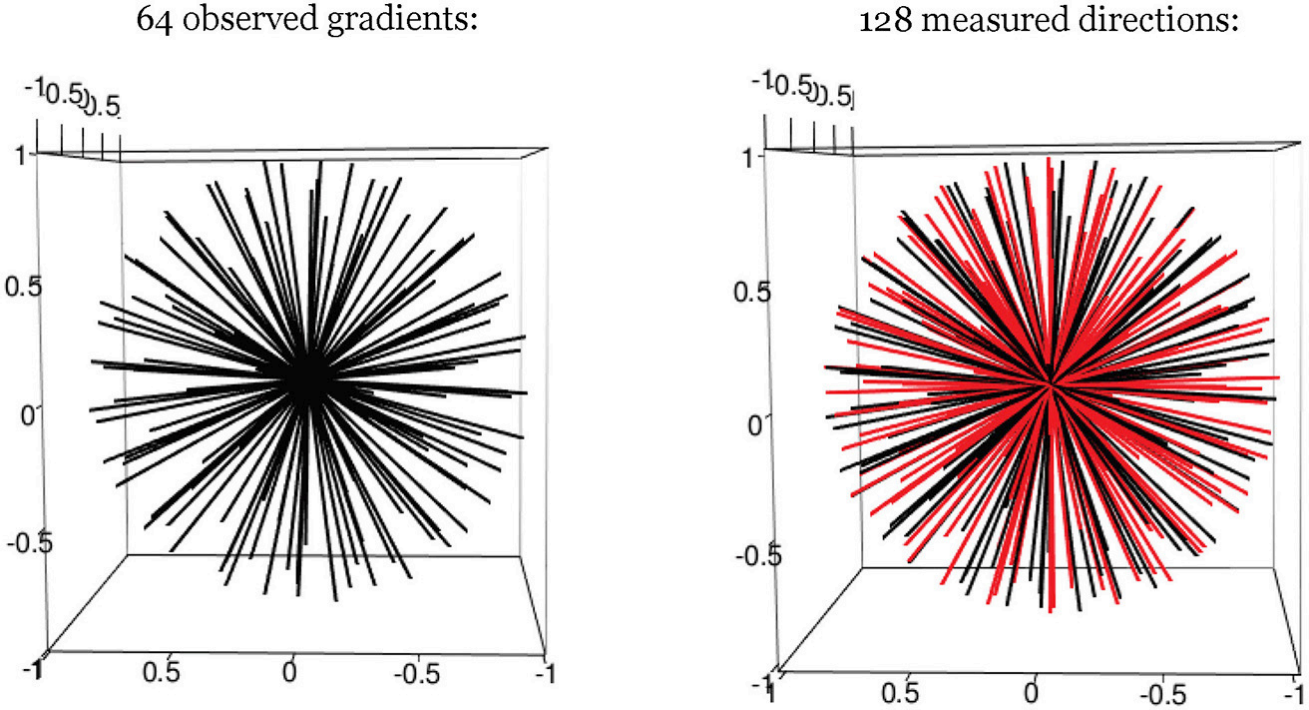
Hypothetical gradient directions. Left: 64 evenly distributed gradient directions; Right: 64 additional hypothetical gradients directions (in red).

One facet of estimation that can be improved with smoothing is the identification of the longitudinal axis, which involves selecting the gradient direction with the largest (smoothed) intensity value. To improve estimation bias that could result from the limited granularity of the observable gradient directions, we add hypothetical gradient directions from which observed data is not actually acquired but estimated through smoothing of observed values. In our cases, this involved 64 “extra” directions, although another number of discrete directions could have been added as well (see Figure 2). The hypothetical directions are generated by the rotation of the whole set of observed gradients, and guided by the principle of maximization of the minimum distance between hypothetical and observed directions. We found for our simulations that the largest angular separation from a gradient to any neighboring direction was < 10°. These hypothetical directions thus expand the estimation space of possible directions for *r*_*l*_, and improve resolution. A smoothed value is obtained as a weighted linear combination of all the observed data. The weights vary and depend on the angular separation between a given (hypothetical) direction, and the other observed directions around it. This is determined by the relative density values of the von-Mises distribution, with a given concentration parameter κ (see Equation 6). As κ decreases, the spread of the distribution is larger, indicating that the neighboring signal intensity data are assigned more weight when smoothing.

By the definition of r_*i*_ and Equation (4), we can readily derive Equation (7) as the basis for obtaining $\hat{r_{l}}$. The estimator $\hat{r_{l}}$ is determined as the direction of the maximal smoothed signal value among all the observed and hypothetical gradient directions. The accuracy of estimator r_*i*_ does rely on selection of the smoothing kernel.

(7) $$\begin{array}{r} \hat{r_{l}}=\arg\;max_{r_{j}}\left(S_{j,smooth}\right) \end{array}$$

Following Equation (4), it also is of interest to accurately distinguish the maximum intensity value itself, and not just the longitudinal axis direction associated with it. Note that the estimation of diffusivity (*d*) and sum of fiber volume fraction (Σ* f*_*k*_) depends on the maximum intensity value in Equation (4). This is a second facet in estimation that can be improved with smoothing.

To note, the estimators on the magnitude of maximum signal (max(*S*)) and the orientation of maximum signal (r_*i*_) appear to, respectively have improved accuracy by choosing different concentration parameters for smoothing, even though both estimators are related to the maximum signal value. To differentiate the smoothing parameters, we denote this second von-Mises smoothing kernel with kappa parameter κ_2_.

#### Guidance on Smoothing Kernel Selection

Extensive simulations have been conducted in Yang (2018) to assess the effect of smoothing kernel selection on nuisance parameter estimation, and more importantly, on fiber-related parameters. These were done assuming 64 gradients and signal to noise ratio of 20. Other signal to noise ratios were considered in Yang (2018), and the results give similar conclusions about relative performance. In summary, a good empirical choice of kappa value, κ, is between 35 and 70 for estimation of max(*S*), which in turn impacts the estimation of *d*, ∑***f***_***k***_. In our simulation analysis, we discover that the error between smoothed maximum diffusion data value across gradients, max(*S*_*j, smooth*_), and the true maximum diffusion data value, max(*S*), is < 5% [see (Yang, 2018)]. For estimation of r_*i*_, a good empirical choice of κ_2_ value will be around the range between 10^−4^ and 0.1. Recall that the estimate of r_*i*_ determines the rotation of an estimated hyperplane for two fiber orientations. Accuracy depends on angular separation of the two fibers. For instance, at 90°, estimation of r_*i*_ is highly accurate with κ_2_ = 0.1, with little to no bias and standard deviation of the discrepancy from the true direction of around 1.5° across simulations. For instance, even at 50° of separation, bias is around 2.7 with standard deviation of discrepancy of 5.8. We adopt κ_2_ = 0.1, and consider it a reasonable value across different angular separation scenarios.

#### Adaptive MCMC Estimation of Simplified and Full Ball-and-Stick Models

We implemented the adaptive MCMC estimation algorithm (Green, 1995; Haario et al., 2001) in our simplified model (Equation 5) as follows:

1. Assign a prior distribution for the simplified parameter space Ω = (f_1_, ${\varphi^{\prime }}_{1}$, ${\varphi^{\prime }}_{2}$, σ). The prior distribution of the noise term σ is non-informative: σ^−2^ ~ Inverse-Gamma (α = 200, β = 1), where α is the shape parameter, β is the scale parameter. Others are non-informative priors: f_1_ ~ U(0,1), ${\varphi^{\prime }}_{1}$ ~ U(0, π), ${\varphi^{\prime }}_{2}$ ~ U(0,π).
2. Iteratively generate parameter values (Ω_1_, Ω_2_, …, Ω_t−1_, Ω_t_) by sampling f_1_, ${\varphi^{\prime }}_{1}$, ${\varphi^{\prime }}_{2}$ from their proposal distributions, until convergence to stationary distribution is attained according to stopping criteria.
3. Update f_1_, ${\varphi^{\prime }}_{1}$, ${\varphi^{\prime }}_{2}$ with a Metropolis-Hastings sampling step. For each of these parameters, a proposal distribution q(Y| Ω_t−1_) (see Equation 8) is used to generate a new candidate parameter value Y. Ω_t_ is then set to Y with a probability α(Ω_t−1_, Y) = min(1, $\frac{\pi \left(Y\right)\;}{\pi \left(\Omega_{t-1}\right)}$) (e.g., π(*Y*) is posterior likelihood given Y, the other most recent parameter values, and observed diffusion data). Otherwise, reject the value Y, and set Ω_t_ to the previous value.
  (8) $$\begin{array}{r} q\left(Y|\Omega_{t-1}\right)=\frac{1}{\sqrt{2\pi \varepsilon^{2}}}\exp\left(-\frac{{\left(Y-\Omega_{t-1}\right)}^{2}}{2\varepsilon^{2}}\right) \end{array}$$
4. Update σ^−2^ with a Gibbs sampling step. The updating of σ^−2^ posterior distribution is conditional on updated values of f_1_, ${\varphi^{\prime }}_{1}$, ${\varphi^{\prime }}_{2}$.
5. The proposal variance ε^2^ for each parameter is adapted every 50 iterations, according to the acceptance rate among those iterations (< 0.44, then decrease ε by the factor of δ; > 0.44, increase ε by the factor of δ. Note that δ = min[exp(0.01), exp(1/c)], where c is the square root of the modulus of t by 50) (Green, 1995).
6. After the MCMC iterations have finished, f_2_ is derived as Σ* f*_*k*_ minus f_1_, and (0, ${\varphi^{\prime }}_{\text{k}}$) is reformulated back into (θ_k_, φ_k_) by re-rotating the angular coordinates to the original coordinate system, guided by the estimate of r_*i*_.

With the full Ball-and-Stick model, we implemented the approach in Behrens et al. (2007) that also fully aligns both methods. This includes adopting the same prior specifications across models when applicable, and the same adaptive MCMC framework. This facilitates comparison between the simplified and full models in terms of computational performance and estimation accuracy of the fiber-specific parameters.

We record the ongoing chains of sampled values until stopping, and the collection of these sampled values can be reflected in histograms and time-series plots (e.g., see Appendix). Estimates of posterior medians and standard deviations are obtained from sampled values after the burn-in period. These estimates will be used to describe the posterior distributions of the parameters of interest (e.g., f_1_, f_2_, ${\varphi^{\prime }}_{1}$, ${\varphi^{\prime }}_{2}$) when evaluating the accuracy and precision of estimates.

## Results

### Simulation Results of Fiber-Specific Parameters

The performance of estimation on the fiber-specific parameters in two crossing fiber scenarios will now be compared between the simplified model and the full model. Again, for the simplified model, the estimated values of the non-fiber-specific parameters *S*_0_*, d*, Σ* f*_*k*_ will be substituted in. To assess the accuracy of estimation of fiber-specific parameters in a MCMC framework, we extract every 10th sampled values from the last half of MCMC chains with 100,000 iterations. For each scenario of parameters being studied, 100 data sets are simulated. Estimates are derived from each data set, so that distributions of these estimates can be generated. Distributions of bias in the posterior median-based estimates can thus be visualized for comparison with the corresponding ground-truth parameter values. Again, we set ground-truth model parameters to be *S*_0_ = 400, *b* = 1500 s/m^2^, *d* = 1/1500 m^2^/s, f_1_ = 0.4, f_2_ = 0.5, θ_1_ = 0, θ_2_ = 0, ϕ_1_ = 60°, ϕ_2_ = 120°. We also set κ_2_ = 0.1. The number of observed gradient directions is 64, as in Jones et al. (2002).

#### Results for Volume Fractions of Individual Fibers

**Figure 3:**
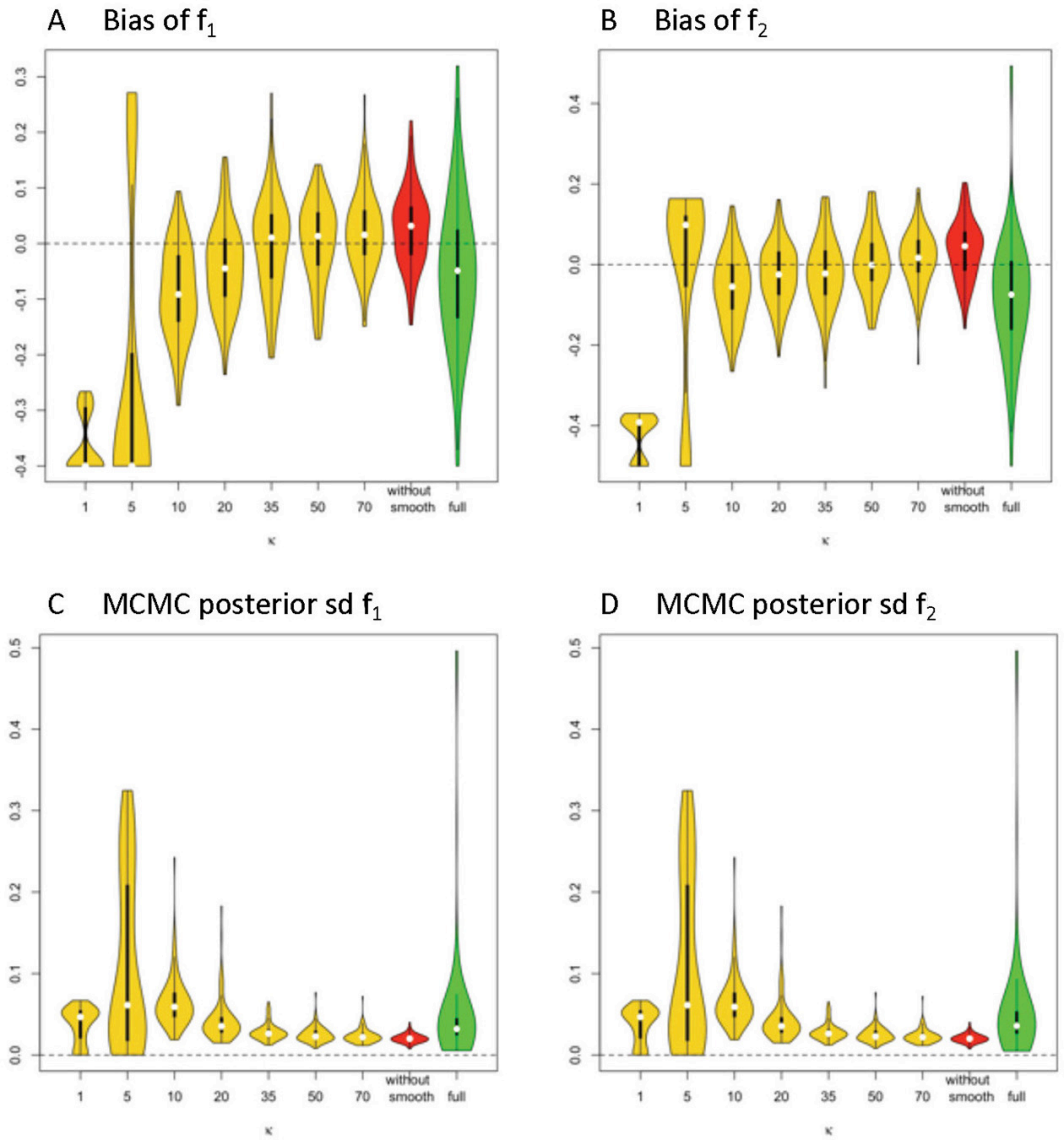
Estimation bias and posterior precision of f_1_ and f_2_ in the violin plot across 100 simulations. **(A)** bias of f_1_, **(B)** bias of f_2_, **(C)** posterior sd of f1, and **(D)** posterior sd of f_2_. Note: *S*_0_ = 400, *b* = 1500 s/m^2^, *d* = 1/1500 m^2^/s, f_1_ = 0.4, f_2_ = 0.5, θ_1_ = 0, θ_2_ = 0, ϕ_1_ = 60°, ϕ_2_ = 120°.

In Figures 3A,B, the violin plot (Hintze and Nelson, 1998) displays the accuracy level of f_1_ and f_2_ under different model settings. Violin plots are a combination of a box plot and a kernel density plot. Specifically, it starts with a box plot, where the thick bar represents the IQR of the bias with the white point “median” in the middle, and the thin bar “whiskers” are drawn to 1.5 × IQR below the 1st quartile or above the 3rd quartile. It then adds a rotated kernel density plot to each side of the box plot.

Generally, the distribution of bias that is centered around 0 and with relatively small variance has a higher accuracy level. The violin plots in yellow and red display the distribution of bias of parameter estimates (medians) of f_1_ and f_2_ in the simplified models, based on estimates of Σ* f*_*k*_ that are derived from different smoothing kernels (i.e., different κ values). The plot in green shows the related distributed bias in the full model. When the κ value is in the range of ≥20, the bias and variance of estimation error appear smaller in the simplified model than the full model. In Table 1, the estimation bias (mean ± sd) for f_1_ and f_2_ demonstrates a more accurate estimate in the simplified model 0.0026 ± 0.0719 and 0.0054 ± 0.0788, respectively (κ = 50), while the corresponding bias is −0.053 ± 0.1344 and −0.0842 ± 0.1496 in the full model.

**Table 1:** Fiber-specific parameters (f_1_, f_2_, θ_1_, ϕ_1_, θ_2_, ϕ_2_) and angular separation bias of two fibers in 64 gradient directions across 100 simulations.

		**Estimation bias (mean ± sd)**			
**Model setting**		**f_**1**_**	$\varphi_{1}^{*}$	$\theta_{1}^{*}$	Angular bias of fiber 1^*^
Simplified	κ = 1	−0.3639 ± 0.0531	26.8 ± 19.6	0.3 ± 2	32.2 ± 8.1
	κ = 5	−0.2534 ± 0.2521	28.9 ± 9.9	0.1 ± 1.1	29.5 ± 7.9
	κ = 10	−0.0874 ± 0.0817	6.3 ± 10.8	0.1 ± 2	10.5 ± 7
	κ = 20	−0.0411 ± 0.078	2.4 ± 9.9	−0.1 ± 3.2	8.2 ± 6.8
	κ = 35	−0.0038 ± 0.0872	0.8 ± 11	0.3 ± 2.9	8.7 ± 7.4
	κ = 50	0.0026 ± 0.0719	−2.3 ± 12	0.4 ± 3.3	9.4 ± 8.5
	κ = 70	0.0202 ± 0.0691	−2.1 ± 9.1	0.8 ± 2.9	7.3 ± 6.5
	Without smoothing	0.0265 ± 0.0682	−4.5 ± 11.2	0.1 ± 1.7	8.7 ± 8.5
Full		−0.053 ± 0.1344	1.7 ± 11.3	3.7 ± 4.3	9.6 ± 8.3
		**Estimation bias (mean ± sd)**			
**Model setting**		**f**_**2**_	$\varphi_{2}^{*}$	$\theta_{2}^{*}$	Angular bias of fiber 2^*^
Simplified	κ = 1	−0.4231 ± 0.0534	−18.9 ± 12.4	0.1 ± 1.4	21.7 ± 6.4
	κ = 5	−0.0292 ± 0.2471	−21 ± 11.6	0.1 ± 0.9	23.5 ± 5
	κ = 10	−0.057 ± 0.0852	−6 ± 6.4	0 ± 0.7	7.4 ± 4.8
	κ = 20	−0.0248 ± 0.0744	−3.1 ± 7.5	0 ± 1.5	6.2 ± 5.5
	κ = 35	−0.0202 ± 0.088	1 ± 9.4	0.1 ± 1.7	7.2 ± 6.4
	κ = 50	0.0054 ± 0.0788	−0.5 ± 8	−0.1 ± 2.1	6.5 ± 5.1
	κ = 70	0.0156 ± 0.0684	1.2 ± 7.9	−0.1 ± 1.4	5.5 ± 5.9
	Without smoothing	0.0351 ± 0.0721	1.1 ± 8	0 ± 0.4	5.9 ± 5.4
Full		−0.0842 ± 0.1496	0.6 ± 9.1	2.7 ± 1.6	7.7 ± 5.6

* *Angular bias is in degrees*.

In Figures 3C,D, the violin plot displays the precision level of f_1_ and f_2_ under different model settings. Posterior standard deviation in the simplified model (κ ≥ 20) is smaller compared with that in the full model. In Table 1, the posterior distributions of f_1_ and f_2_ demonstrate a more precise estimate in the simplified model 0.0252 ± 0.0094 (κ = 50), while the corresponding estimation bias is 0.054 ± 0.0835 and 0.0585 ± 0.0833, respectively in the full model.

**Figure 4:**
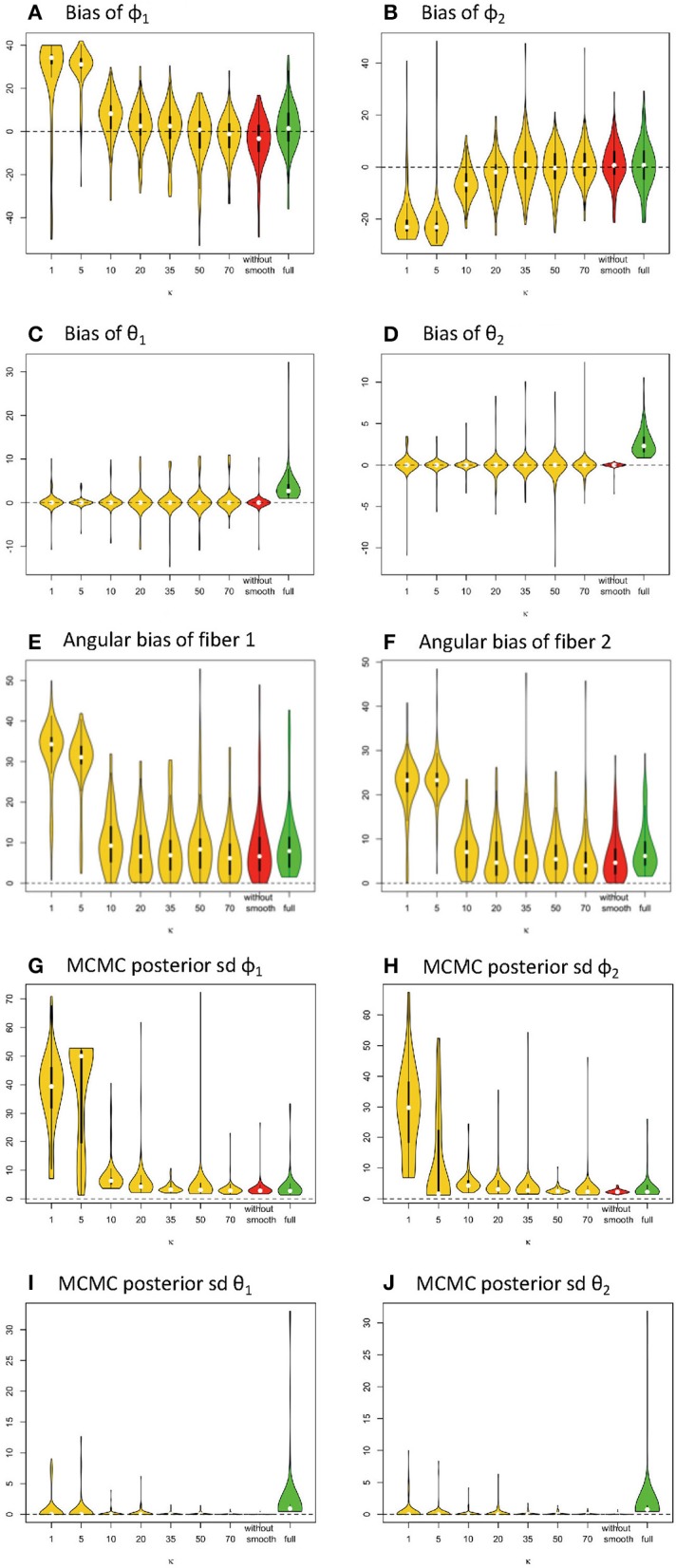
Estimation bias and posterior precision of fiber orientations (in degrees) in the violin plot with 64 gradients across 100 simulations. **(A)** bias of φ_1_, **(B)** bias of φ_2_, **(C)** bias of θ_1_, **(D)** bias of θ_2_, **(E)** angular bias of fiber 1, **(F)** angular bias of fiber 2, **(G)** posterior sd of φ_1_, **(H)** posterior sd of φ_2_, **(I)** posterior of θ_1_, and **(J)** posterior of θ_2_. Note: *S*_0_ = 400, *b* = 1500 s/m^2^, *d* = 1/1500 m^2^/s, f_1_ = 0.4, f_2_ = 0.5, θ_1_ = 0, θ_2_ = 0, ϕ_1_ = 60°, ϕ_2_ = 120°.

Among the simplified models, we noticed that the performance in estimation of f_1_ (Figure 4A) and f_2_ (Figure 4B) is closely related to the performance of estimator of Σ* f*_*k*_, in that a more biased estimate of Σ* f*_*k*_ will lead to a more biased estimate of f_1_ and f_2_. Across varying κ values, the estimates of volume fractions with respect to κ equal to 50 obtain the smallest bias and variance, which is consistent with the empirical suggestions for κ value selection in the estimation of max(S), d, and ∑***f***_***k***_. Similarly, beyond 60 degree fiber angular separation, we also found the estimation of f_1_ and f_2_ with κ equal to 50 generally performs at least as well as those for other κ values. Generally, when the fiber angular separation is larger, the estimation of parameters is “easier,” so that the estimates of f_1_ and f_2_ are more accurate and precise. For instance, when the angular separation between fibers reaches its largest value of 90°, the estimation bias of f_1_ and f_2_ has smallest mean (relative to 0) and standard deviation.

#### Results for Orientations of Individual Fibers

Similarly, in Figure 4, the violin plots in yellow and red display the distribution of bias from parameter estimates (medians) of θ_1_, φ_1_, θ_2_, φ_2_ in the simplified models, derived from different smoothing kernels (e.g., different κ values). The plot in green shows the related distribution of bias in the full model (Figures 4A–D). We define *angular bias* as the difference in estimated and true fiber direction in their shared hyperplane. Note that this measure of bias incorporates information about the estimation of both θ, φ. We assess angular bias of fiber estimates in Figures 4E–F and Table 1.

In comparison of the results, the simplified and full models demonstrated a similar accuracy level of performance in estimating the fiber orientations (Figures 4E,F and Table 2). Bias in estimated fiber orientations for the simplified model are very close or slightly better than for the full model, when the κ value is ≥20. The performance of estimates for angular separation of two fibers indicates similar results between the simplified model and the full model (see Table 1). Similarly, beyond the scenario of 60° fiber angular separation, we also found comparable patterns between some simplified model setting and the full model under other scenarios (e.g., 90°, 30° fiber angular separation). The simplified model demonstrates a similar precision level of performance in estimating the fiber orientations (Figures 4G–J and Table 2), in that the MCMC posterior standard deviation on fiber orientations in the simplified model are also very close or slightly better than the full model, when the κ value is at least 20.

**Table 2:** Posterior standard deviation of the fiber–specific parameters (f_1_, f_2_, θ_1_, ϕ_1_, θ_2_, ϕ_2_) in 64 gradient directions across 100 simulations.

		**Posterior sd (mean ± sd)**		
**Model setting**		**f_**1**_**	$\varphi_{1}^{*}$	$\theta_{1}^{*}$
Simplified	κ = 1	0.0375 ± 0.0214	37.7 ± 13.1	0.8 ± 2.2
	κ = 5	0.1121 ± 0.1062	37.5 ± 18.4	0.5 ± 2.2
	κ = 10	0.0651 ± 0.0312	8 ± 6.3	0.1 ± 0.5
	κ = 20	0.0422 ± 0.0256	5.6 ± 6.8	0.2 ± 0.7
	κ = 35	0.0282 ± 0.01	3.7 ± 1.6	0.1 ± 0.3
	κ = 50	0.0252 ± 0.0094	4.2 ± 7.1	0.1 ± 0.2
	κ = 70	0.0231 ± 0.0084	3.2 ± 2.2	0 ± 0.1
	Without smoothing	0.0205 ± 0.0054	3.2 ± 2.5	0 ± 0.1
Full		0.054 ± 0.0835	4.1 ± 5.4	2.3 ± 5.8
		**Posterior sd (mean ± sd)**		
**Model setting**		**f**_**2**_	$\varphi_{2}^{*}$	$\theta_{2}^{*}$
Simplified	κ = 1	0.0375 ± 0.0214	28.9 ± 14.3	0.5 ± 1.6
	κ = 5	0.1121 ± 0.1062	11.9 ± 15.3	0.2 ± 1.1
	κ = 10	0.0651 ± 0.0312	5.4 ± 3.4	0.1 ± 0.5
	κ = 20	0.0422 ± 0.0256	4.2 ± 4.7	0.2 ± 0.7
	κ = 35	0.0282 ± 0.01	3.5 ± 5.2	0.1 ± 0.3
	κ = 50	0.0252 ± 0.0094	2.7 ± 1.1	0.1 ± 0.2
	κ = 70	0.0231 ± 0.0084	3 ± 4.5	0.1 ± 0.2
	Without smoothing	0.0205 ± 0.0054	2.4 ± 0.6	0 ± 0.1
Full		0.0585 ± 0.0833	3.5 ± 4.4	2.1 ± 5.8

*Standard deviations are in degrees.

#### Computational Efficiency of MCMC Simulation

The computation of simulation analysis was run on a server computer with the following: CPU Intel Xeon E5-2699 v5 32 cores, 128 GB RAM, Linux-based CentOS 7 environment. In terms of the computing efficiency, the simplified model required an average of approximately 0.65 min per 100,000 iterations as in 3.5.3, when the computation of 20 simulated samples was run simultaneously over 10 cores. The full model required an average of approximately 9.5 min per 100,000 iterations under the same conditions. The simplified model thus offers a great reduction in computing times.

### Real Data Examples and Three Fiber Simulated Scenarios

**Figure 5:**
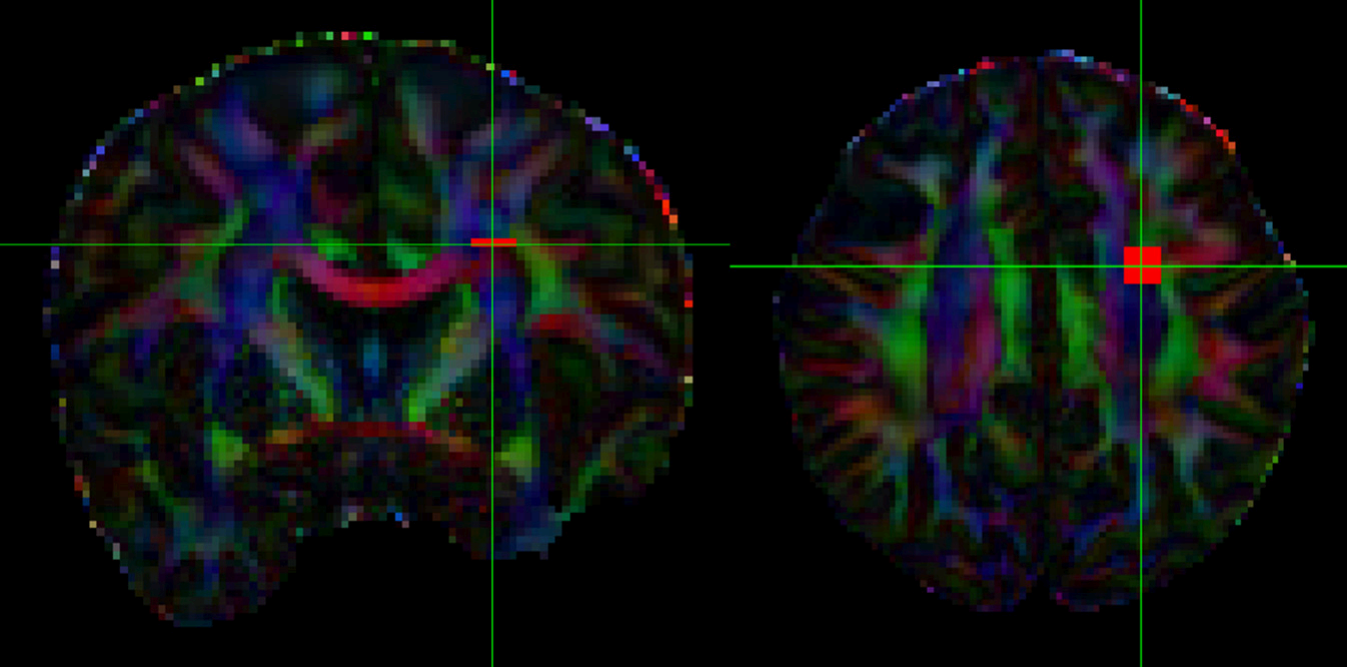
Region of interest in the corona radiata. The orange square indicates the voxels.

To obtain an *in-vivo* human data example, we used freely available neuroimaging data that was acquired by the MGH HCP team for the Human Connectome Project (https://db.humanconnectome.org/data/projects/MGH_DIFF). The study subject ID is MGH 1001, a female aged between 40–44 years old. A data analysis was conducted in FSL following their processing pipeline guide, using BET, EDDY_CORRECT, DTIFIT, and BEDPOSTX. In order to evaluate the full and simplified models with real data, we selected an area of 36 contiguous voxels in the corona radiata region as test samples. The corona radiata is an area that contains many crossing fibers. See Figure 5, which indicates location of the region of interest being analyzed.

**Figure 6:**
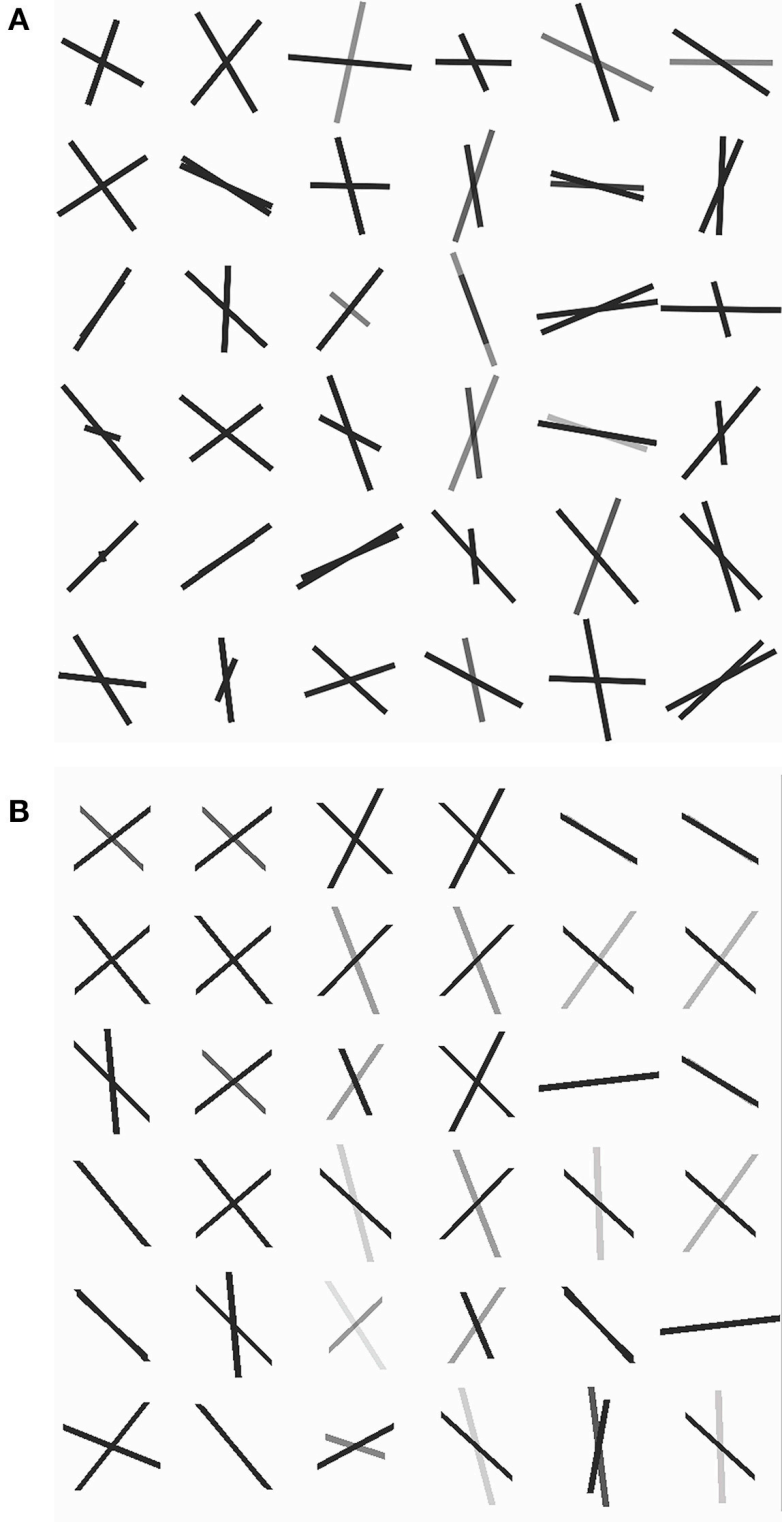
Dyadic vectors demonstrating the angular orientations between estimated θ_1_, φ_1_, and θ_2_, φ_2_. from real data. **(A)** Simplified model estimates, **(B)** BEDPOSTX model estimates.

To assess how our 2-fiber simplified model behaves in such situations, we also estimated 3-fiber models using BEDPOSTX for comparison. To indicate the presence of 3 fibers, we examined the volume fraction of the third fiber (f_3_) across the entire brain and selected the 99th percentile value (f_3_ = 0.1) as the threshold for indicating the presence of the third fiber. According to this criterion, 20 of the 36 voxels were likely to contain 3 fibers. As there is no gold standard for real data, we simply present the results (see Supplementary Table 1, where specific voxels are identified, and voxel-level estimates are listed). We also compute respective angular differences between the first two estimated directions of the 3-fiber BEDPOSTX model and the two directions estimated by the simplified model. See Figure 6, where the voxels are in the region of interest of Figure 5. For the apparent 3-fiber voxels, the median angular and volume fraction f-value difference in estimates for the first of the directions are 10.25° and 0.014; for the second direction they are 11.30 and 0.033. For the apparent 2-fiber voxels, estimated median angular and f-value differences are 2.87° and 0.0070, and 4.89° and 0.0086. Overall, median differences with the first two angles and f-values estimated by the 3-fiber BEDPOSTX model and the simplified model are generally not large. For the one-fiber voxel in BEDPOSTX (voxel 52 86 61), the angular difference for the respective angle estimated with the simplified model is 9.00°, and difference in estimated f_1_ values is 0.01. The simplified model estimated f_1_ and f_2_ as 0.47 and 0.23, so with this model, there is indication of a second angle. For the no-fiber voxel indicated by BEDPOSTX (voxel 51 83 61), the simplified model estimates f_1_ and f_2_ as 0.18 and 0.17. Generally, Figure 6 likely reflects the anterior-posterior alignment of underlying fibers.

We also considered three simulated 3-fiber scenarios, 1,000 replications each with signal-to-noise ratio equal to 5% of S_0_ = 400:

1. φ_1_ = 0, θ_1_ = 0, φ_2_ = 90, θ_2_ = 0, φ_3_ = 45, θ_3_ = 45; f_1_ = 0.3, f_2_ = 0.25, f_3_ = 0.2.
2. φ_1_ = 25, θ_1_ = 25, φ_2_ = 0, θ_2_ = 0, φ_3_ = 25, θ_3_ = 0; f_1_ = 0.4, f_2_ = 0.25, f_3_ = 0.1.
3. φ_1_ = 0, θ_1_ = 0, φ_2_ = 90, θ_2_ = 0, φ_3_ = 90, θ_3_ = 90; f_1_ = f_2_ = f_3_ = 0.25.

We used the BEDPOSTX 3-fiber model and simplified 2-fiber model for estimation. Given estimated directions from each method, we considered all permutations of pairing the estimated directions with the true fiber directions in the respective simulations, and selected the permutation with smallest average angular difference between paired estimated and true directions. Angular and f-value differences, as well as the number of permutations for the simplified model that a true direction is associated with an estimate, are listed in Table 3. Note that for both methods, the 3-fiber scenarios that were studied present difficult estimation problems, and that bias in estimation is large. An exception is in Scenario 3, where the third direction is consistently estimated in a fairly accurate manner by the simplified model.

**Table 3:** Summary of simulation results, with angular distance and volume fraction differences across 1000 simulations.

	**Angle 1 distance**	**Angle 2 distance**	**Angle 3 distance**	**f_**1**_ difference**	**f_**2**_ difference**	**f_**3**_ difference**
**SCENARIO 1-SIMPLIFIED**						
Median	73.2°	75.4°	44.7°	0.045	0.109	0.106
Inter-quartile range (IQR)	5.5°	6.6°	3.3°	0.045	0.055	0.055
Number of Permutations/1000	159	841	1000			
**SCENARIO 1—BEDPOSTX**						
Median	78.6°	86.9°	85.0°	−0.023	0.035	0.040
IQR	54.9°	50.1°	79.8°	0.174	0.330	0.317
Number/1000	1,000	1,000	1,000			
**SCENARIO 2—SIMPLIFIED**						
Median	69.3°	62.2°	78.8°	−0.126	0.325	0.397
IQR	28.1°	24.1°	23.9°	0.339	0.246	0.357
Number/1000	1,000	80	920			
**SCENARIO 2**—**BEDPOSTX**						
Median	38.1°	107.7°	99.0°	−0.400	−0.241	−0.099
IQR	93.4°	86.2°	91.3°	0.415	0.665	0.651
Number/1000	1,000	1,000	1,000			
**SCENARIO 3—SIMPLIFIED**						
Median	70.1°	63.7°	6.5°	−0.015	−0.010	−0.016
IQR	39.0°	38.5°	10.9°	0.045	0.050	0.046
Number/1000	458	542	1,000			
**SCENARIO 3-BEDPOSTX**						
Median	71.8°	70.8°	43.2°	−0.249	−0.249	−0.249
IQR	43.0°	44.4°	60.9°	0.064	0.062	0.061
Number/1000	1,000	1,000	1,000			

### Impact of Increased Gradient Directions (64 vs. 128 Gradients)

A simulation study was implemented to demonstrate how the increase in the number of observations from gradient directions impacts the estimation results. This has practical importance, as it is expected in the near future for standard clinical DWI acquisitions to have increased number of gradients. We simulated a set of 128 evenly distributed gradient directions and followed the same estimation procedures and parameter settings to obtain the parameter estimates as we did previously with simulations with 64 gradient directions. The performance of estimation on the fiber-specific parameters is compared between 128 vs. 64 gradient directions, respectively between the simplified model and the full model.

**Figure 7:**
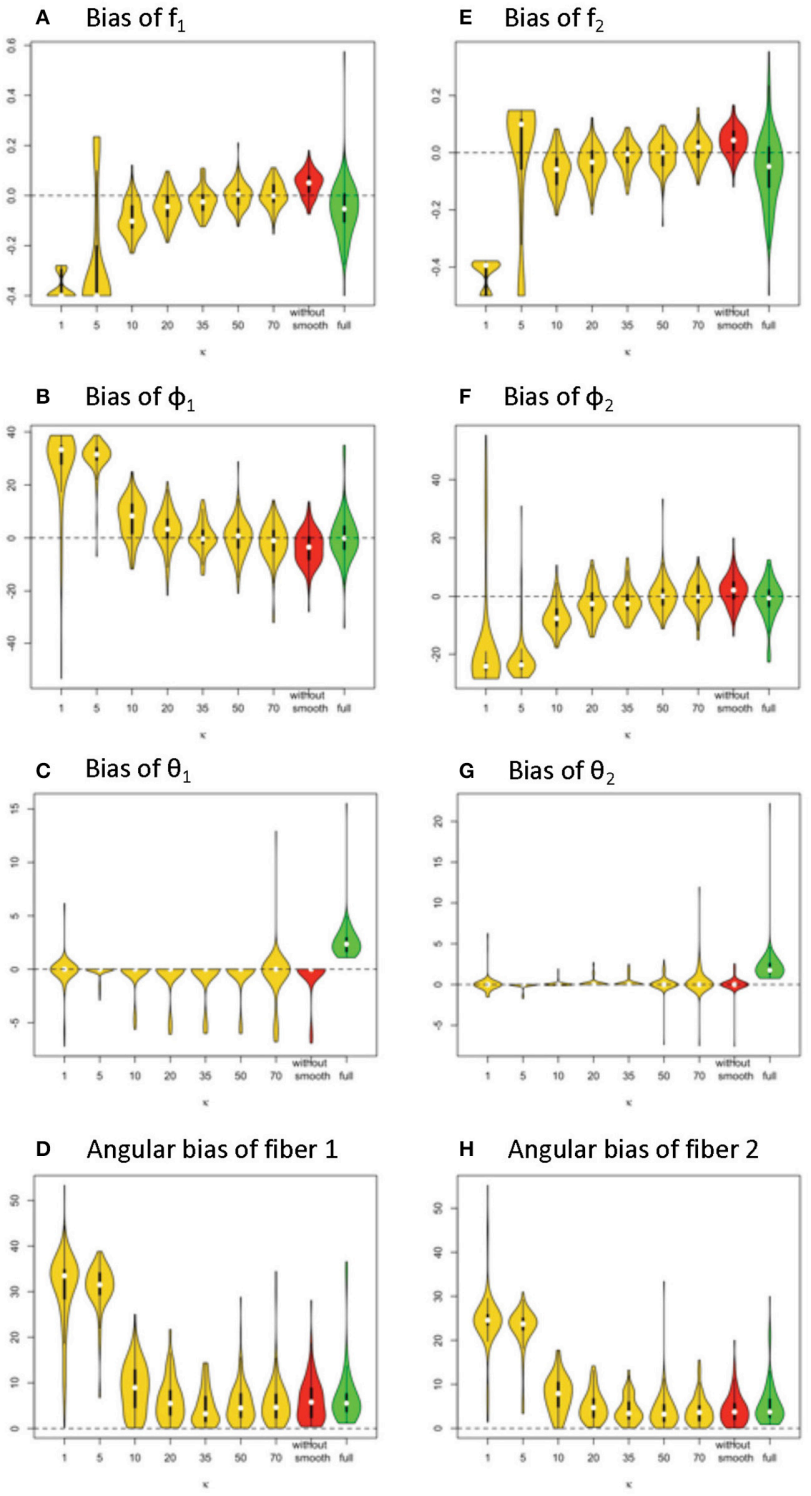
Estimation bias of fiber volume fractions and orientations of two fibers in the violin plot (128 gradient directions) across 100 simulations. **(A)** bias of f_1_, **(B)** bias of f_2_, **(C)** bias of φ_1_, **(D)** bias of φ_2_, **(E)** bias of θ_1_, **(F)** bias of θ_2_, **(G)** angular bias of fiber 1, and **(H)** angular bias of fiber 2. Note: *S*_0_ = 400, *b* = 1500 s/m^2^, *d* = 1/1500 m^2^/s, f_1_ = 0.4, f_2_ = 0.5, θ_1_ = 0, θ_2_ = 0, ϕ_1_ = 60°, ϕ_2_ = 120°.

Given 128 gradients, the bias and variance of fiber-related estimates are smaller in the simplified model than the full model, when the κ value or estimating is in the range of 20 or higher (see Figure 7). Within the results from the simplified model, the estimates of volume fractions with respect to κ between 35 and 70 is likely to obtain the smallest bias across varying κ values. When the value of κ was chosen as 20, 35, 50, 70 or even without smoothing, the estimation error on volume fractions in the simplified model was generally closer to 0 than that in the full model.

In terms of fiber orientation, the simplified model demonstrates a similar level of performance in estimation. The biases of estimates of fiber orientations in the simplified model are very close and slightly improved relative to the full model when the κ value was 20 or higher. Similarly, as with the trend with volume fraction estimation error, the estimates of fiber orientations with respect to κ between 35 and 70 obtain the smallest bias across varying κ values.

Comparing estimation bias with 128 vs. 64 gradients (Table 4 vs. Table 1), biases tend to decrease with 128 directions across different model settings. To note, in terms of angular bias estimation on fiber 1, a decreased mean by 4° and a decreased standard deviation by 4° in the simplified model (κ = 50) indicates a more accurate and precise estimate. While bias also is smaller in estimating the full model with more observations from added gradients, the magnitude of improvement was not as great as with the simplified model (κ = 50). Within the results of the simplified model, an empirical suggestion on the choice of κ value is between 35 and 70 to obtain smaller bias and standard deviation of estimation error, no matter if the number of gradients is 128 or 64 (Tables 1, 4).

**Table 4:** Estimation bias of the fiber-specific parameters (f_1_, f_2_, θ_1_, φ_1_, θ_2_, φ_2_) and angular bias of two fibers in 128 gradient directions across 100 simulations.

		**Estimation bias (mean ± sd)**			
**Model setting**		**f_**1**_**	**φ_1_**	**θ_1_**	**Angular bias of fiber 1**
Simplified	κ = 1	−0.3678 ± 0.0507	27.3 ± 15.9	−0.2 ± 1.5	30.5 ± 8.3
	κ = 5	−0.2698 ± 0.2343	30.7 ± 5.9	−0.2 ± 0.6	30.8 ± 5.1
	κ = 10	−0.09 ± 0.0668	7.2 ± 7.3	−0.5 ± 1.4	8.8 ± 5.4
	κ = 20	−0.0435 ± 0.0602	3 ± 7	−0.6 ± 1.7	6.2 ± 4.7
	κ = 35	−0.0237 ± 0.0509	0.2 ± 5.8	−0.7 ± 1.9	4.7 ± 3.9
	κ = 50	−0.0004 ± 0.0494	0.1 ± 6.9	−0.6 ± 1.7	5.4 ± 4.6
	κ = 70	0.0047 ± 0.0506	−1.5 ± 7.1	−0.8 ± 2.5	5.7 ± 5.1
	Without smoothing	0.0462 ± 0.0487	−3.9 ± 6.5	−0.6 ± 1.8	6.1 ± 4.8
Full	−0.0527 ± 0.1122	0.4 ± 9.1	2.6 ± 1.6	7 ± 6.5
		**Estimation bias (mean ± sd)**			
**Model setting**		**f**_**2**_	**φ_2_**	**θ_2_**	**Angular bias of fiber 2**
Simplified	κ = 1	−0.4227 ± 0.0498	−17.9 ± 18.1	0.1 ± 1.1	24.6 ± 6.6
	κ = 5	−0.0193 ± 0.2331	−21.7 ± 7.8	−0.1 ± 0.4	22.6 ± 4.8
	κ = 10	−0.0614 ± 0.0657	−7 ± 5.2	0.1 ± 0.3	7.7 ± 4.1
	κ = 20	−0.0359 ± 0.0598	−2.2 ± 5.7	0.2 ± 0.5	5 ± 3.3
	κ = 35	−0.0108 ± 0.0483	−1.8 ± 4.9	0.2 ± 0.6	4.3 ± 3
	κ = 50	−0.0081 ± 0.0538	0.2 ± 5.6	0.1 ± 1	4 ± 4
	κ = 70	0.0132 ± 0.049	0.2 ± 5	0.4 ± 1.6	4.1 ± 3.2
	Without smoothing	0.0397 ± 0.0483	1.9 ± 5.2	0.1 ± 0.9	4.4 ± 3.5
Full	−0.0564 ± 0.1253	−1.3 ± 6.6	2.1 ± 2.2	5.5 ± 4.9

As we investigate other challenging estimation scenarios with two crossing fibers (e.g., fiber angular separation of 40°), we have seen that the MCMC chains in the simplified model have a much higher likelihood of accurate convergence with 128 gradients. The simplified model is clearly more stable in such scenarios.

### Dynamic Stopping Rule in MCMC Estimation

The MCMC algorithm has served as a powerful approach for Bayesian estimation of DWI modeling. This method involves simulating from a complex and generally multivariate target distribution with respect to the parameters in the models. Markov chains are generated and stationary distributions of sampled parameter values are targeted (Cowles and Carlin, 1996). In application of MCMC algorithms, a default number of iterations can be overly large to guarantee a sufficient length for burn-in period, and to attain stationary convergence. However, this can result in an unnecessary computation load. This issue is compounded due to the large number of voxels. We thus suggest applying convergence criteria such as the Geweke diagnostic (Green, 1995) to dynamically detect the stationary convergence along Markov chains, which provides guidance on when to stop sampling at the voxel level. Further technical discussion and an example that illustrates great computational savings through reduced number of iterations for MCMC are given in the Appendix. An important practical implication is that the simplified model not only requires less computation for a given set of iterations, as was seen earlier, but also requires a lesser number of iterations. In conjunction, these computational savings are multiplicative.

### Model Selection: 1-Fiber vs. 2-Fiber Simplified Model

We also have considered how to identify 1-fiber vs. 2-fiber model fit within a voxel during a same MCMC run. This is similar in purpose as the Automatic Relevance Determination (ARD) sparsity prior in Behrens et al. (2007), which identifies whether to continue to include fiber components within a same MCMC run. An advantage of these approaches is that model fit can be conducted without having to conduct MCMC separately per model. We can efficiently identify sampling chain behaviors that indicate over-fitting of a 2-fiber model with 1-fiber data without the need to add to model complexity, as ARD priors do. One-fiber models converge in MCMC estimation quite quickly relative to 2-fiber models, so in conjunction with dynamic stopping of MCMC sampling at the voxel level, computation can be reduced even further. Details are given in the Appendix.

## Discussion

The key step for our simplified model is Equation (5), which allows for focus on fiber-specific parameters that reduce the parameters in the non-linear regression that are to be estimated in a MCMC framework. Non-fiber-specific parameters can be estimated reliably through simultaneous equations for estimation that mainly take advantage of novel statistics based on signal shape across gradient acquisitions. Specifically, the estimated maximum (max(*S*)) and spherical mean ($\overset{\bar{}}{S}$) of signal shape allow for a practical and valid solution for estimating the parameter *d* and Σ*f*_*k*_, and for estimating the longitudinal axis (r_*i*_) of the signal surface shape. The latter is used to guide the rotation of the hyperplane of two fiber orientations to further reduce angular parameterization. Spatial smoothing of intensity values improves estimation performance. After the replacement with the non-fiber-specific parameter estimators, only three parameters are then to be estimated in the MCMC framework under the scenarios of two crossing fibers.

The results of fiber-specific parameter estimation consistently demonstrate the simplified model approach be more accurate, precise and efficient. From our extensive simulations across different data sets, we see that fiber parameter estimation with the proposed approach is apparently unbiased, or close to unbiased. Nuisance parameter estimation also is apparently unbiased with relatively small error (Kaden et al., 2016). Simulations of the complete two-stage procedure give us a sense of the variability in estimation, in terms of the resultant fiber parameter point estimates from Bayesian analysis (e.g., Figures 4, 5). Since the proposed estimation process is (close to) unbiased and can have less variability, it is relatively attractive. First, the estimates of volume fraction f_1_ and f_2_ in a simplified model setting (e.g., with smoothing parameters κ = 50, κ_2_ = 0.1) can always outperform that in the full model setting regardless of the angular separation Δφ_fibers_, in that the mean bias can be slightly reduced and the precision level can improve by more than 2-fold (smaller standard deviation of estimation error). Similarly, the estimates of fiber orientation can also be more accurate and precise in a proper simplified model. Importantly, we observed that the computational run time for the simplified model can approximately be 14–15 times faster than that in the full model for the same number of MCMC iterations. This does not even take into account that the convergence within a single chain is much faster in the simplified model, in terms of needing a fewer number of iterations before convergence is attained. This simplified model can require up to 10 times less iterations for stopping. With dynamic stopping of MCMC chains per voxel, these reductions in computation times are multiplicative. The issue of computational efficiency is critical as current probabilistic fiber tracking that relies on voxel-level MCMC estimation of the full Ball-and-Stick model is extremely slow and time-consuming.

Since we are conditioning on the estimated nuisance parameter values, the Bayesian estimation process for the fiber parameters does not reflect the uncertainty in nuisance parameter estimation. In terms of using the posterior distributions for probabilistic fiber tracking, we do note that the median values of the resultant posterior distributions will often be close to the associated true value, due to the apparent lack of bias in point estimation. Smaller posterior variances will lead to more of the sampled values being close to the true value as well, and reduced variability in fiber tracking. So, we think it will be attractive to conduct probabilistic fiber tracking with the proposed MCMC-generated posteriors distributions.

The simplified model still inherits the limitation from the full model in that it cannot reliably estimate two crossing fibers that are not far apart in terms of angular separation when the number of gradient directions is limited. For instance, when two fibers are 40° apart (Δφ_fibers_ = 40°), the angular bias is around 15 ± 10 (mean and standard deviation from 100 simulations) given 64 gradients; however, the bias is 8 ± 7 given 128 gradients. The number of gradients in an MR diffusion image acquisition thus has an obvious impact on the accuracy and precision of parameter estimation, as well as extending the feasibility of estimation when fiber directions are not as well separated. In the near future, 128 gradient scans will be standard in clinical acquisitions. Based on our simulations, fiber direction estimation based on the simplified model holds promise for even greater relative precision improvements as the number of gradients increases.

The proposed approach clearly does not have the capability to model 3-way crossings, even if it can sometimes estimate one or two of the directions accurately in such situations. Still, the proposed approach can be useful if used in conjunction with 3 fiber models, such as in an adaptive “step down” model, once 3-way crossings can be ruled out. We illustrate such an adaptive “step down” approach from two-fiber to one-fiber models and show computational savings in that setting. Nonetheless, 3-fiber modeling can be very difficult even when explicitly acknowledging 3 fibers in a model, as illustrated in the simulations presented here.

Although not explored here, this approach can be extended to multi-shell data. One possible approach with multi-shell data could be to estimate the *d* and Σ* f*_*k*_ parameters in parallel based on respective samples with a specific b-value, and then pooling the estimates, such as by respectively, averaging them. The MCMC estimation algorithms can then be implemented as in section Adaptive MCMC estimation of simplified and full Ball-and-Stick models, while recognizing different b-values as in Equation (1).

## Conclusion

In summary, a simplified version of the Ball-and-Stick model is proposed. By reducing the parameter space dimensionality in the non-linear regression estimation framework, the computing time in the simplified model can be shortened dramatically. We believe the overall time savings will be tremendous as we transition to implementation at the whole brain level. Meanwhile, the accuracy and precision of estimating the fiber volume fractions and fiber orientation can also be improved with less complex and numerically simpler non-linear regressions. Future consideration will be given to Laplace approximations of the posterior distributions associated with fiber parameters, which is more feasible with the reduced dimensionality. We will also explore whether some of the ideas applied here can be extended to 3-fiber models, to reduce parameterization of corresponding non-linear regression models.

## Ethics Statement

The human subject data used in this study was obtained from a freely available MRI database, the Human Connectome Project. Their study was conducted according to Good Clinical Practice guidelines and the Declaration of Helsinki. Ethical approval was obtained from their Institutional Review Board before commencing subject enrolment. Written informed consent was obtained from all subjects and/or their authorized representatives before protocol-specific procedures were carried out. Ethics approval from our local ethics committee was not required to analyze this anonymized dataset.

## Author Contributions

SY: study design, analysis, interpretation of data, preparation of manuscript. KG: study design, interpretation of data, preparation of manuscript. KS: study design, interpretation of data, preparation of manuscript. SS: study design, preparation of manuscript. SC: interpretation of data, preparation of manuscript. CT: study design, analysis, interpretation of data, preparation of manuscript.

### Conflict of Interest Statement

The authors declare that the research was conducted in the absence of any commercial or financial relationships that could be construed as a potential conflict of interest. The reviewer JDT declared a shared affiliation, with no collaboration, with one of the authors, SC, to the handling editor at time of review.
