# Supplementary material for: A Simplified Crossing Fiber Model in Diffusion Weighted Imaging
Source: Front Neurosci. 2019 May 22;13:492. doi: 10.3389/fnins.2019.00492 (PMC6541109; doi:10.3389/fnins.2019.00492)
Supplement: Supplementary file 2 [file Data_Sheet_1.docx]

**Appendix**

**A.1 Dynamic stopping rule in MCMC estimation**

In (1), Geweke recommended using methods from spectral analysis to assess convergence, for each univariate parameter Θ being simulated (here, Θ indicates any individual parameter of f_1_, f_2_, φ′_1_, φ′_2_) (2). This approach relies on a t-test to assess the equality of the means of the first and last sections of a Markov chain. More specifically, Geweke’s convergence diagnostic after n iterations is calculated by taking the difference between the means $\bar{\Theta}_{n}^{A}$, based on the first n_A_ iterations, and means $\bar{\Theta}_{n}^{B}$, based on the last n_B_ iterations, and dividing by the asymptotic standard error based on both subsamples. If the ratio n_A_/n and n_B_/n are held fixed and n_A_ + n_B_ < n, then by the central limit theorem, the distribution of this diagnostic statistic Z approaches a standard normal as n tends to infinity (see Equation A1). Geweke suggested using n_A_ = 0.1 × n and n_B_ = 0.5 × n.

$Z={(\bar{\Theta}_{n}^{A}-\bar{\Theta}_{n}^{B})}/{\sqrt{\frac{\sigma_{A}^{2}}{n_{A}}+\frac{\sigma_{B}^{2}}{n_{B}}}}$ (Equation A1)

We can apply this Geweke diagnostic dynamically to separately monitor for stationary convergence in the respective chains of all the parameters being sampled in the MCMC framework (f_1_, φ′_1_, φ′_2_). Evidence of convergence will be assessed in a systematic, repeated manner (e.g. every 1,000 iterations) across parameters. The stopping rule decides to terminate the MCMC procedure for a given voxel for instance when the corresponding Geweke statistic for each parameter has p-value less than 0.05/*k*, *k* = number of parameters.

In our analysis, we can obtain a graphical review of the MCMC convergence, by scrutinizing the histogram and time-series plot of the posterior samples. To illustrate, the full chains of sampled posterior values of interest from MCMC in the full model and those in the simplified model are investigated, given the ground-truth model parameters set as S_0_ = 400, b = 1500, d = 1/1500, f_1_ = 0.2, θ_1_ = 0, φ_1_ = 55°, f_2_ = 0.7, θ_2_ = 0, φ_2_ = 125°, σ = 5% of S_0_. In the full model, the estimation results show that the Markov chains of estimation tend to attain stationary convergence around the 200,000th iteration in the time-series plot (note that every sample for summary inference is extracted by a thinning interval of every 10 iterations, as in the plot). In contrast, the estimation results suggest that the posterior chains in the simplified model have reached convergence up to 10 times faster, by requiring only a run length of approximately 20,000 iterations. In our simulation, the number of iterations between assessments of the Geweke diagnostics for determining whether to stop the voxel-level MCMC was set to 1,000 iterations.

**A.2 Model Selection: 1-fiber vs. 2-fiber simplified model**

An adaptive method of model selection was developed in our study. The strategy of this method is to start model fitting with the 2-fiber model, and adaptively step down to 1-fiber model conditionally based on observation of the features that indicate an over-specification of a fiber in the 2-fiber model fit. Importantly, our approach will allow for one model fitting MCMC run, as opposed to two separate model fits, with a comparison step such as Bayes factors. Behrens et al. (3) used an Automatic Relevance Determination (ARD) prior to identifying redundant components from the model. However, this approach requires more modeling complexity and sampling steps, which we are trying to minimize for the sake of computational efficiency.

Note that the 1-fiber model is a nested model with respect to the 2-fiber model, with the constraint that one of volume fractions (f_1_ or f_2_) is equal to 0. In this case, either f_1_ or ${\sum f}_{k}$ - f_2_ would be equal to 0 as shown in Equation 5, and therefore the angle with respect to the volume fraction of value 0 is disassociated with the intensity value.

Technically, when 1-fiber data is fit with a 2-fiber model, evidence of an over-specified fiber model can be observed in the corresponding MCMC chains: 1) the posterior distribution of the volume fraction of the over-specified fiber converges to point mass on 0; 2) the corresponding sampled posterior angles will vary widely. As an example approach for quantitatively capturing these two features of an over-specified fiber, the criteria of switching to 1-fiber model can be: 1) 80^th^ percentile of volume fraction of one of the fibers is essentially equal to 0, and less than a threshold value of say 10^-3^_._ 2) The circular variance of the same fiber in Condition 1 is greater than 0.8. Note that the circular variance, Var(φ), 1 minus the mean resultant length of the unit vectors, is the complement to the mean resultant length (η) of all the unit vectors of angles (Equation A2). As for interpretation, note that when the circular variance is 0, this indicates that all angles are in the same direction; it is equal to 1 if angles are distributed uniformly across possible circular values; 3) Conditions 1 and 2 occur together in a predetermined sliding window length that establishes consistency in chain behavior.

$Var\left( \phi\right)=1-\eta$ (Equation A2)

where

$$\eta=\frac{1}{m}\sqrt{(\sum_{n=1}^{m} \cos\phi_{m})^{2}+(\sum_{n=1}^{m} \sin\phi_{m})^{2}}$$

with *m* being the number of samples covering the length of a sliding window.

Motivation for these conditions is as follows. Condition 1 indicates that most posterior values are close to 0. Condition 2 indicates that the estimated fiber values are varying wildly. Condition 3 helps insure that we are not observing spurious results. We do acknowledge that these conditions are exploratory, and that other similar conditions can provide identification as well. We next consider simulations of one-fiber and two-fiber scenarios, and assess identification of over-specification. We assess our identification rules for over-specification of one-fiber data with a two-fiber model. We also consider one-fiber models that may actually be adequate when two-fiber data has low angular separation. We only study the simplified model here.

**Simulation Results**

In simulation analyses, we set up three sets of simulated data and fit them into 2-fiber simplified models separately, aiming to discover the behavior of estimation and the performance of our detection rules given one of the fibers is over-specified (f_1_ = 0) or have minor volume fraction values (f_1_ = 0.05 or 0.1). When evidence of over-specification is shown in 2-fiber fit, we would fit the data into 1-fiber for comparison. The other fiber is assumed to have a larger volume fraction (f_2_ = 0.5). Thus, we vary the ground-truth model parameters f_1_ to be 0, 0.05, 0.1 to generate three sets of data, and meanwhile other parameters in the voxel are set as S_0_ = 400, b = 1500 s/m^2^, d = 1/1500 m^2^/s, f_1_ = 0.5, θ_1_ = 0, θ_2_ = 0, φ_1_ = 60°, φ_2_ = 120°. In the simulation results, if f_1_ estimate is greater than f_2_ estimate, we switch the labeling of two fibers, in order to align with the constraint f_2_ > f_1_ in simulated model setting (in Figure A1).

We first fit 1-fiber simulated data (f_1_ = 0, f_2_ = 0.5) with 2-fiber simplified models. Figure A1 displays histogram and time-series plots, which illustrate the posterior distributions of fiber-specific parameters under 2-fiber model fit.

**Figure A1.** Estimation performance with 2-fiber and 1-fiber simplified model (f_1_ = 0). Note: Parameter values for simulated data: S_0_ = 400, b = 1500, d = 1/1500, f_1_ = 0, θ_1_ = 0, φ_1_ = 60°, f_2_ = 0.5, θ_2_ = 0, φ_2_ = 120°, σ = 5 % of S_0_. Unit of angles is in degrees.

A) Posterior f_1_ in 2-fiber model (after burn-in):


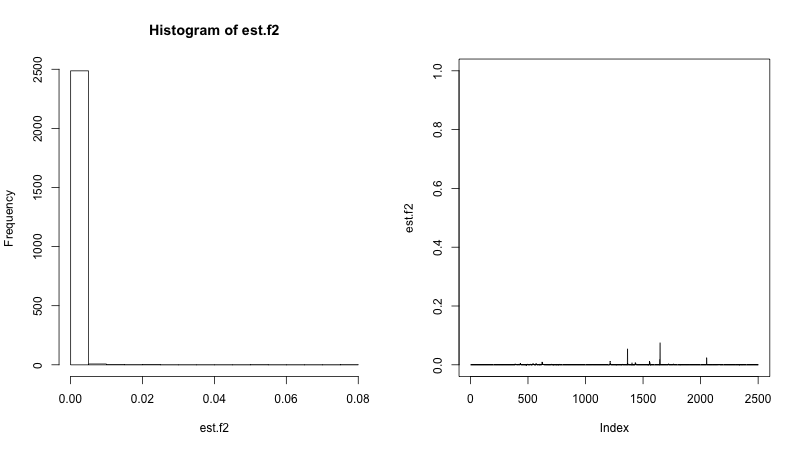


B) Posterior f_2_ in 2-fiber model (after burn-in):
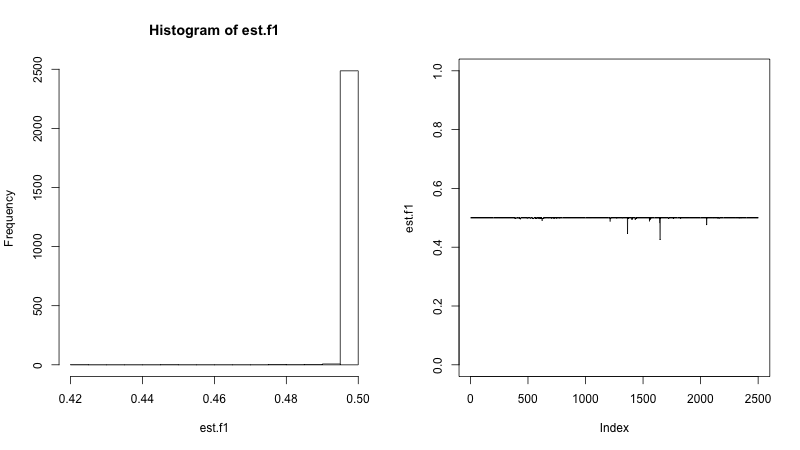


C) Posterior φ′_1_ in 2-fiber model (after burn-in):
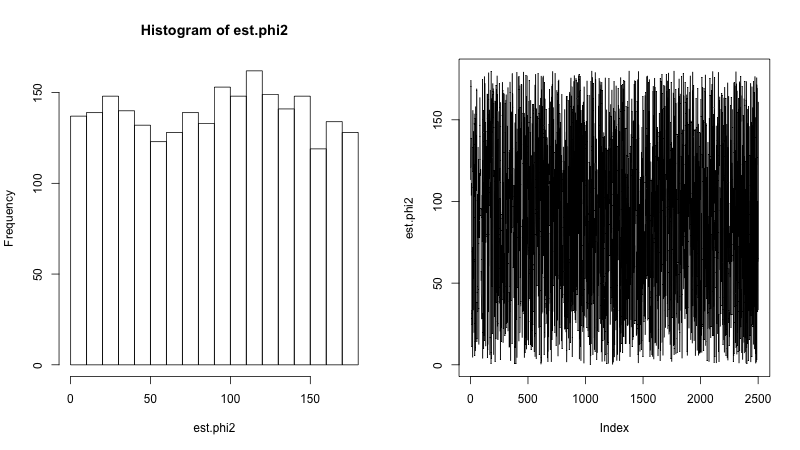


D) Posterior φ′_2_ in 2-fiber model (after burn-in):


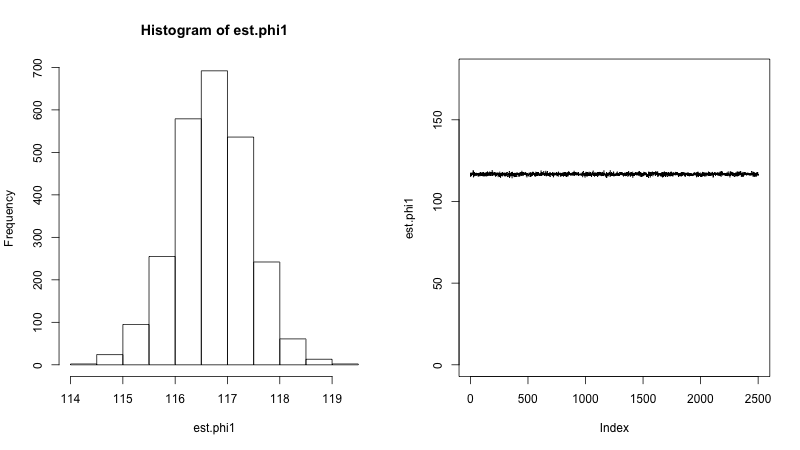


E) Posterior φ′_1_ in 1-fiber model (full chain):


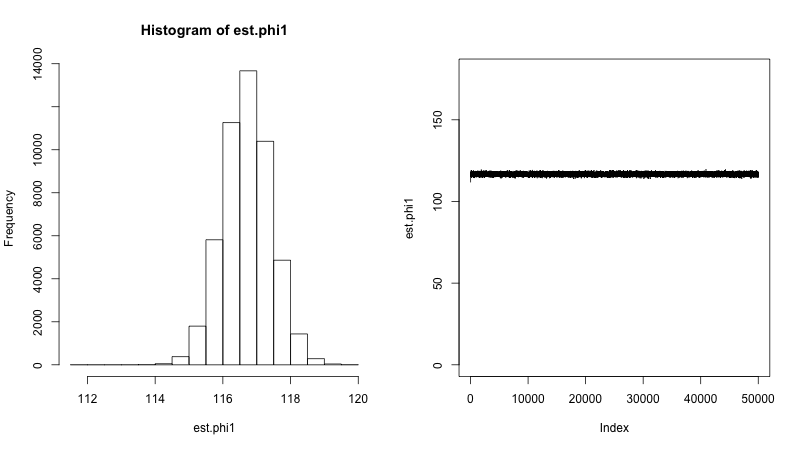


After observing fiber model over-specification, we consider terminating the dynamic MCMC iterations after a sufficient length (condition 3 of over-specification: e.g. over a sliding window of 750 iterations), and transitioning to 1-fiber model fitting. The 1-fiber simplified model is a much more parsimonious model, since there is only a single parameter estimation (fiber orientation) in the MCMC framework. In Figure A1-(E), the 1-fiber model fit appropriately estimates the orientation of major fiber (φ_2_ = 120°), and the full chain of posterior φ_2_ converges extremely fast.

Following that, another two sets of simulation are analyzed (smaller volume fraction values equal to 0.05 or 0). This is in order to investigate the sensitivity of the identification of fiber over-specification in cases when the value of a volume fraction in the two-fiber model is closer to 0. We set ground-truth model parameter value of f_1_ to be 0.05, and fix the other parameters to be same magnitude as with the previous simulated data. In Figure A2, the simulation with f_1_ equal to 0.05 shows a similar result of fiber over-specification as that with f_1_ = 0 in Figure A1-(E). The conditions of over-specified fiber can be identified in the posterior chains of f_1_ and φ′_1_. Further, note that as seen in Figure A2, the one-fiber model can still estimate the major fiber tract angle fairly accurately.

**Figure A2.** Estimation performance with 2-fiber and 1-fiber simplified models (f_1_ = 0.05). Note: Parameter values for simulated data: S_0_ = 400, b = 1500, d = 1/1500, f_1_ = 0.05, θ_1_ = 0, φ_1_ = 60°, f_2_ = 0. 5, θ_2_ = 0, φ_2_ = 120°, σ = 5 % of S_0_. Unit of angles is in degrees.

A) Posterior f_1_ in 2-fiber model (after burn-in):


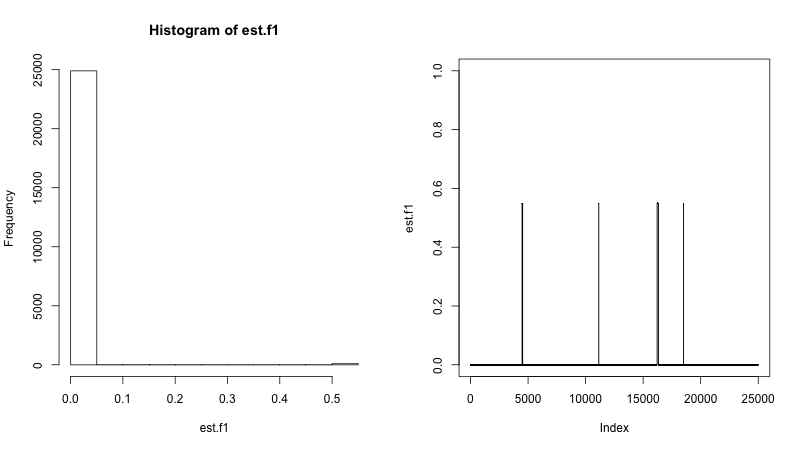


B) Posterior f_2_ in 2-fiber model (after burn-in):


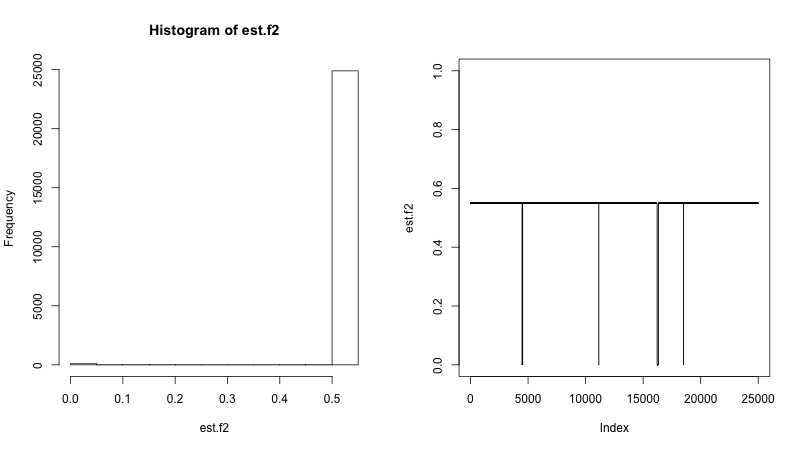


C) Posterior φ′_1_ in 2-fiber model (after burn-in):


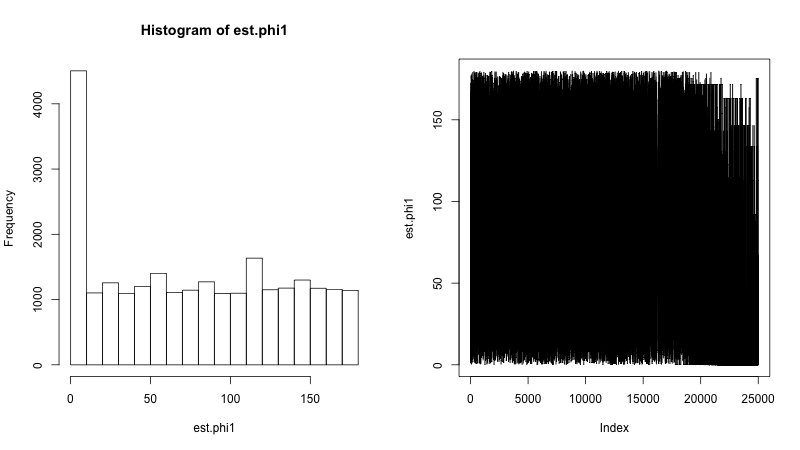


D) Posterior φ′_2_ in 2-fiber model (after burn-in):

^
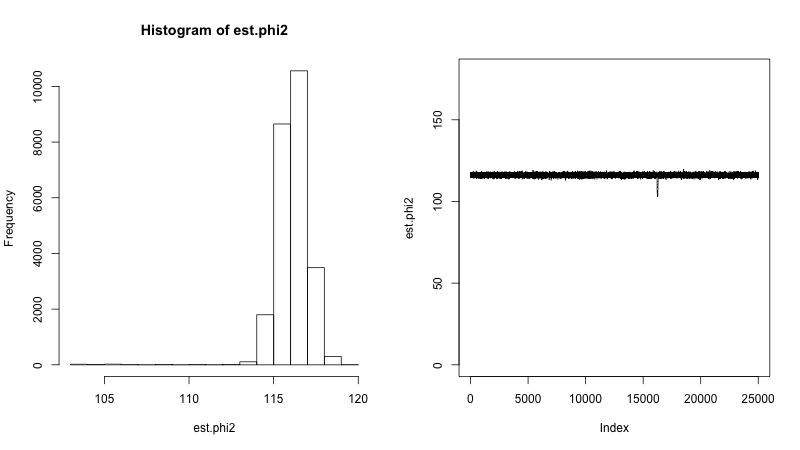
^

E) Posterior φ′_1_ in 1-fiber model (full chain):


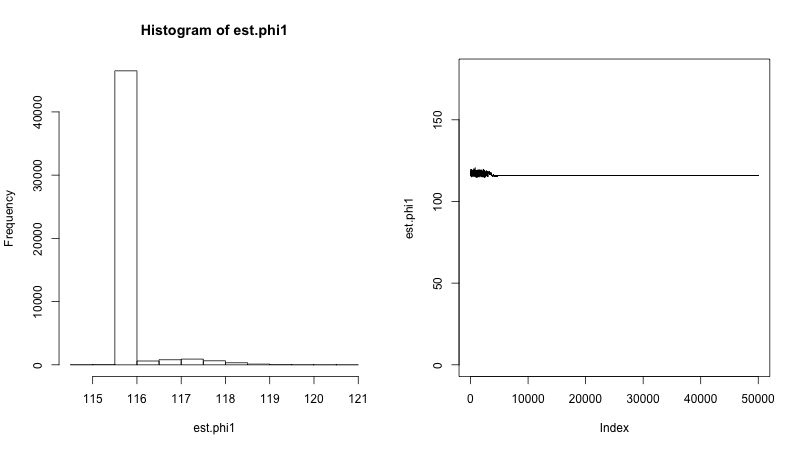


Lastly, we set the ground-truth model parameters f_1_ to be 0.1 and fix the other parameters to be the same within the previous simulated data. In Figure A3, the estimation of f_1_ when it is equal to 0.1 does not provide indication of fiber over-specification, which differs from the simulations with smaller volume fraction value of 0 or 0.05 (Figure A1 and Figure A2). The result gives a demarcation for when estimation related to two fibers is feasible, and when all the fiber-specific parameters f_1_, f_2_, φ_1_ and φ_2_ converge accurately in the posterior chains.

**Figure A3.** Estimation performance with 2-fiber simplified model (f_1_ = 0.1). Note: Parameter values for simulated data: S_0_ = 400, b = 1500, d = 1/1500, f_1_ = 0.1, θ_1_ = 0, φ_1_ = 60°, f_2_ = 0.5, θ_2_ = 0, φ_2_ = 120°, σ = 5 % of S_0_. Unit of angles is in degrees.

A) Posterior f_1_ in 2-fiber model (after burn-in):


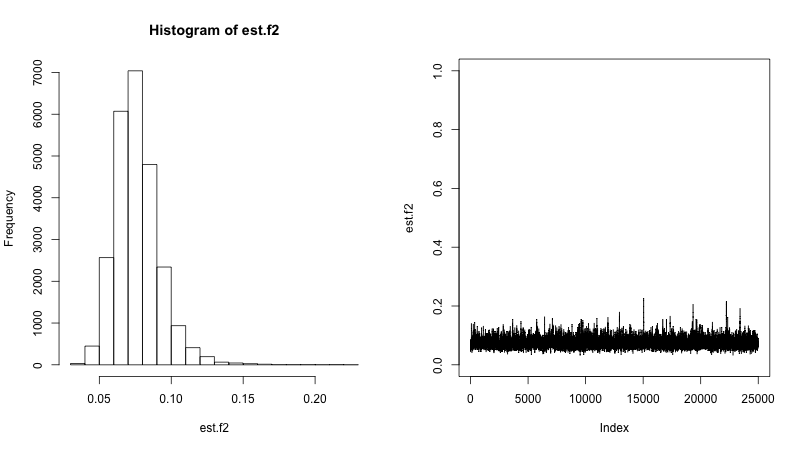


B) Posterior f_2_ in 2-fiber model (after burn-in):


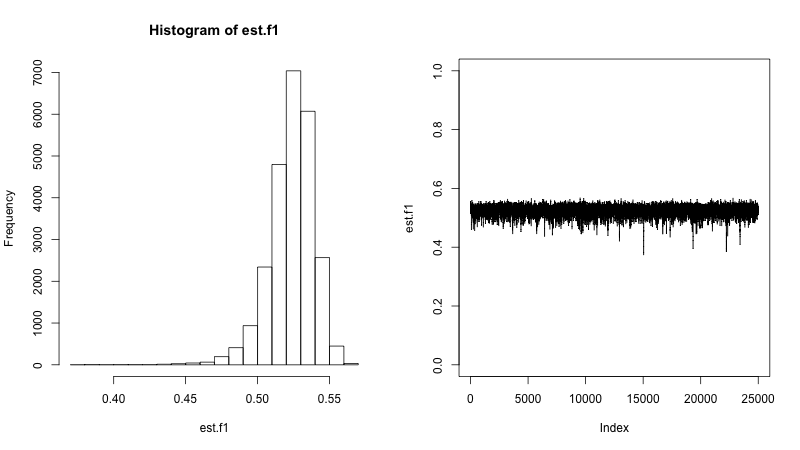


C) Posterior φ′_1_ in 2-fiber model (after burn-in):
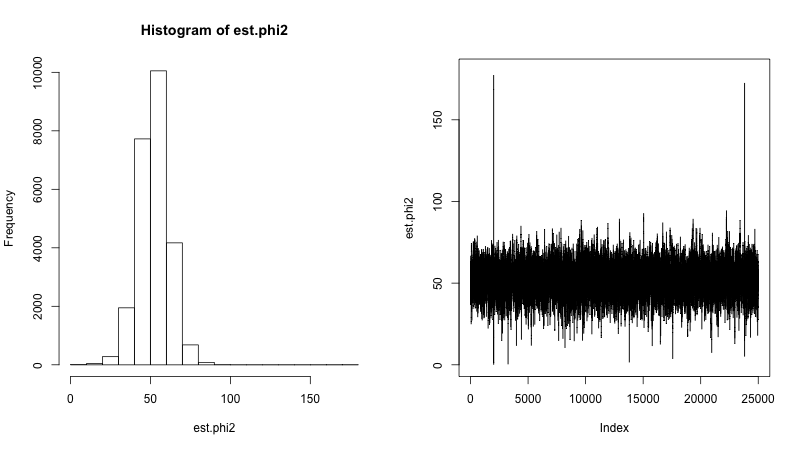


D) Posterior φ′_2_ in 2-fiber model (after burn-in):


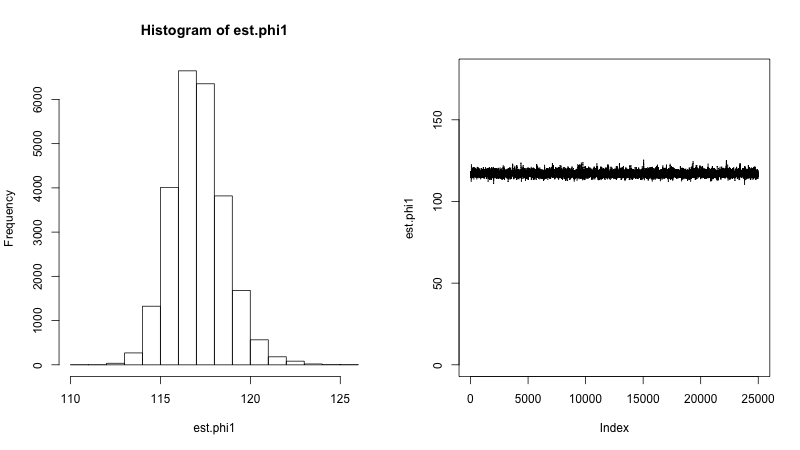


In summary, an empirical boundary on estimation feasibility for smaller volume fraction values in the 2-fiber simplified model fits could be at about 0.10. When one of the volume fractions is too small (e.g. f < 0.05), the 2-fiber simplified model may fail to properly estimate the parameters of the minor fiber.

We applied the adaptive method of model selection in our simulation study. To illustrate the performance of this method, we used the simulated 1-fiber data of parameters f_1_ = 0, f_2_ = 0.5, φ_2_ = 135° with the 2-fiber model fit, and observed that the technique of adaptive model selection can dynamically identify the conditions of fiber over-specification, and effectively terminate the 2-fiber model fitting during a same MCMC run. In our simulation, the tail of the posterior for f_1_ converges to the value of 0, and posterior of φ_1_ spreads wildly with a large corresponding circular variance. The method leads to improved efficiency, with only 4,250 iterations required for stopping.

**Appendix References**

1. J. Geweke: Evaluating the Accuracy of Sampling-based Approaches to the Calculation of Posterior Moments. In: *Bayesian Statistics 4*. Oxford University Press, Oxford (1992)

2. M. K. Cowles and B. P. Carlin: Markov Chain Monte Carlo Convergence Diagnostics: A Comparative Review. *Journal of the American Statistical Association*, 91(434), 883-904 (1996) doi:10.2307/2291683

3. T. E. J. Behrens, H. J. Berg, S. Jbabdi, M. F. S. Rushworth and M. W. Woolrich: Probabilistic Diffusion Tractography with Multiple Fibre Orientations: What can we gain? *Neuroimage*, 34(1), 144-155 (2007) doi:10.1016/j.neuroimage.2006.09.018
